# Application of transgenic zebrafish for investigating inflammatory responses to nanomaterials: Recommendations for new users

**DOI:** 10.12688/f1000research.128851.1

**Published:** 2023-01-12

**Authors:** Helinor J Johnston, Suzanne L J Gillies, Rachel Verdon, Vicki Stone, Theodore Henry, Lang Tran, Carl Tucker, Adriano G Rossi, Charles R Tyler

**Affiliations:** 1 Nano Safety Research Group, School of Engineering and Physical Sciences, Heriot Watt University, Edinburgh, EH14 4AS, UK; 2 School of Energy, Geoscience, Infrastructure and Society, Heriot Watt University, Edinburgh, EH14 4AS, UK; 3 Institute of Occupational Medicine, Edinburgh, EH14 4AP, UK; 4 Centre for Inflammation Research, University of Edinburgh, Edinburgh, EH16 4TJ, UK; 5 Biosciences, College of Life and Environmental Sciences, University of Exeter, Exeter, EX4 4QD, UK

**Keywords:** nanomaterial, nanotoxicology, zebrafish, neutrophil, inflammation

## Abstract

Despite the increasing exploitation of nanomaterials (NMs) in an array of consumer products, there are uncertainties regarding their potential adverse impact on human health. Investigation of whether NMs activate a pro-inflammatory response is routinely used to assess their toxicity in *in vitro* and *in vivo* (rodent) studies. The use of zebrafish ( *Danio rerio*) to investigate inflammatory responses to chemicals, pathogens and injury has increased considerably over recent years. Zebrafish have also been used to investigate the role of inflammation in disease pathogenesis and for drug discovery. Availability of transgenic strains which express fluorescent proteins in immune cells (e.g. macrophages and neutrophils) enables the visualization and quantification of immune cell accumulation in the target site(s) of interest. We therefore propose that transgenic zebrafish have great utility for screening the toxicity of NMs via investigation of inflammatory responses. Indeed, we have successfully used non-protected life stages of transgenic zebrafish with fluorescent neutrophils (Tg(mpx:EGFP ^114^) to investigate inflammatory responses to NMs. The more widespread use of transgenic zebrafish in nanotoxicology could reduce the reliance placed on rodents and thereby enhance the implementation of the 3Rs principles. As zebrafish continue to grow in popularity it is timely to offer guidance to new users on their use. Here we will reflect on: exposure routes that can adopted  to mimic human/rodent exposure, what transgenic strains and life stages are best suited to investigate inflammatory responses, selection criteria for zebrafish embryos/larvae, the inclusion of appropriate controls, the importance of dose selection and sample size, and how the (inflammatory) response can be quantified. It is hoped that our recommendations will support the development of standard protocols that can be used to assess whether NMs activate inflammatory responses. Importantly, the themes discussed are not restricted to NMs but relevant also to zebrafish application in ecotoxicology or human health focused studies.

Research highlights **Scientific benefits**

• Non-protected life stages of transgenic zebrafish can be used to screen the toxicity of nanomaterials (NMs) via investigation of inflammatory responses.
• Zebrafish could provide better predictions of the impact of substances on human health than rodents

**3Rs benefits**

• The use of non-protected life stages of zebrafish could reduce and/or replace the use of rodents in nanotoxicology

**Practical benefits**

• The inflammatory response to NMs can be monitored in the same zebrafish over time, a major advantage over rodents
• The use of zebrafish can make testing cheaper and quicker

**Current application**

• Zebrafish can be exploited to assess the adverse impact of NMs on human health via investigation of inflammatory responses.

**Potential application**

• A wider panel of NMs should be assessed in the future
• Different administration routes should be considered to better mimic human exposure to NMs
• Different transgenic strains should be explored to further probe the mechanism of NM toxicity
• The model is not restricted to nanotoxicology and should be exploited more widely by other disciplines

## Introduction

Toxicity testing has traditionally relied on the use of rodents when assessing the potential impact of substances on human health, often following test guidelines from the Organisation for Economic Co-operation and Development (OECD). Use of alternative (non-rodent) models is increasing to promote alignment with the 3Rs principles of reduction, refinement and replacement of animal use in scientific research, to make toxicology testing more ethical, quicker and often cheaper. The concern that rodents do not always provide a good prediction of human responses further illustrates the need to find alternative models for toxicology testing ( Hartung, 2009).

Zebrafish ( *Danio rerio*) are a highly versatile vertebrate model used widely for biomedical and (eco) toxicology research ( Lieschke and Currie 2007). A prominent use of zebrafish is in the hazard assessment of chemicals, and in particular for ecotoxicity testing using the validated Fish Embryo Acute toxicity (FET) test which assesses the impact of substances on zebrafish embryo development ( OECD Test Number 236, 2013, Hill *et al*., 2005). Over recent years the use of zebrafish for human health related research has expanded. More specifically, zebrafish have been used to study the pathogenesis of many diseases such as cancer (e.g., Hason and Bartůněk 2019, Letrado *et al.,* 2018, Stewart *et al.,* 2014, Lu *et al*., 2015), cardiovascular disease (e.g. Bournele and Beis 2016, Taylor *et al.,* 2019), neurological diseases (e.g., Fontana *et al.,* 2018, Ünal and Emekli-Alturfan 2019), infectious diseases (e.g., Gomes and Mostowy 2020, Torraca and Mostowy 2018), and inflammatory diseases that affect different organs such as the lungs, intestine and liver (e.g. Renshaw *et al.,* 2007, Brugman, 2016, Zhang *et al*., 2016, Hanyang *et al.,* 2017, Shwartz *et al.,* 2019). In addition, zebrafish are increasingly used to accelerate drug discovery by screening the bioactivity and toxicity of new compounds ( Vliegenthart *et al*., 2014a, MacRae and Peterson 2015, Keßler, Rottbauer and Just 2015, Letrado *et al.*, 2018, Bournele and Beis 2016, Hoodless *et al.,* 2016, Yoganantharjah and Gibert 2017, Stewart *et al.,* 2014, Hill *et al.,* 2005, Horzmann and Freeman 2018).

There are many advantages of using zebrafish over rodents for toxicology testing when investigating the potential impact that substances have on human health. Firstly, studies that have employed zebrafish for toxicology testing have shown good correlation with human data ( Nadanaciva *et al.,* 2013, Sukardi *et al*., 2011, Sipes *et al.,* 2011, Tal *et al.,* 2020). For example, excellent concordance (100%) between zebrafish and humans was observed when assessing drug induced hepatotoxicity ( He *et al.,* 2013). Similarly, zebrafish had a 100% prediction success rate for cardiotoxic drugs in humans ( Zhu *et al*., 2014). In addition, a meta-analysis for >600 chemicals indicated that zebrafish could be used as an initial screen to prioritise chemicals for rodent testing and as a potential replacement for mammalian testing ( Ducharme *et al.,* 2015). Of benefit is that biomarkers for toxic responses can often be measured across different species; for example, the liver-enriched microRNA (miRNA) miR-122 is a highly conserved biomarker for hepatoxicity which can be assessed in zebrafish and could be used to predict hepatotoxicity of substances in humans ( Vliegenthart *et al*., 2014b). Other advantages of using zebrafish over rodents include their small size, relative ease and cost of maintenance compared with rodents, high fecundity, availability of genetically manipulated (transgenic) strains, embryo/larval transparency (e.g. which facilitates direct *in vivo* visualization of developmental processes, and organ/tissue structure and function), and their amenability to high throughput testing. Furthermore, a major advantage associated with the use of zebrafish in toxicology (and other disciplines) is their potential to offer many 3Rs benefits, which are discussed in more detail later. In the EU, early life stages of zebrafish, prior to exogenous (independent) feeding at 120 hours/5 days post fertilisation (hpf or dpf)), are not protected by law and as such do not require permission from responsible authorities to be used in scientific research ( EU Directive 2010/63/EU). However, as adult fish are required to obtain a supply of zebrafish embryos/larvae, then the adult fish would need to be housed in approved facilities.

## Transgenic zebrafish

Both wild type and transgenic strains of zebrafish have been used in existing studies that have a human health focus. The optical transparency of zebrafish embryos and larvae combined with the expression of fluorescent proteins in transgenic zebrafish lines allows real time imaging of cellular processes, organelles, cells and tissues ( He *et al*., 2014, Lee *et al.,* 2015, Bai and Tang 2020, Choe *et al*., 2021). Transgenic zebrafish which express fluorescent proteins have been developed for visualisation of a diverse range of targets including but not limited to immune cells ( Renshaw *et al.,* 2006, Ellett *et al*., 2011), neurones ( Park *et al.,* 2000, Winter *et al*., 2021), blood vessels ( Gao *et al.,* 2016), the liver ( Zhang *et al*., 2014), pancreas ( Huang *et al*., 2001), kidney ( Perner *et al*., 2007), mitochondria ( Kim *et al.,* 2018, Zhou *et al*., 2018), Golgi apparatus ( George *et al*., 2014), oestrogen receptors ( Lee *et al*., 2012, Green *et al.,* 2018), ovaries ( Akhter *et al*., 2016), markers of oxidative stress ( Kusik *et al.,* 2008, Mourabit *et al.,* 2019), and intracellular signalling pathways (e.g. NfkB ( Kanther *et al*., 2011)) (reviewed by e.g. Choe *et al*., 2021, Lee *et al*., 2012).

Knockout strains of zebrafish are also available which allow the role of specific genes to be probed in studies that focus on investigating disease pathogenesis, toxicity and drug discovery. Of particular relevance to this paper is the use of knockout strains for investigating the role of different immune cell types in inflammatory responses; however, the use of knockout strains is not the focus of this paper. Examples include where chemokine (CXC) ligand-8 (CXCL8) knockouts have been used to investigate the role of CXCL8 in neutrophil recruitment ( de Oliveira *et al*., 2013), and the use of knockout strains to demonstrate that mitochondria are important to neutrophil motility *in vivo* ( Zhou *et al.,* 2018). Approaches available to deplete functional macrophages and neutrophils from zebrafish may also be of interest and have been reviewed by Rosowski (2020).

## Zebrafish and inflammation

Components of the immune system of zebrafish are similar to that of humans, with the key cell lineages present including monocytes, tissue macrophages, granulocytes (e.g., neutrophils, eosinophils) and lymphocytes ( Traver *et al*., 2003, Meeker and Trede, 2008, Novoa and Figueras, 2012). The innate immune system is functional within 48 hpf, with the adaptive system developing after ~4 weeks ( Renshaw *et al*., 2006, Trede *et al*., 2004, Novoa and Figueras, 2012). The ability to focus on the innate inflammatory response alone is often considered an additional benefit of using early life stages of zebrafish in biomedical research.

Over the last decade, the use of zebrafish as an alternative to rodents for investigating inflammatory responses to various stimuli (e.g. chemicals, pharmaceuticals, pathogens and injury) has increased considerably. Zebrafish have also been used to investigate the contribution of inflammation to disease pathogenesis. A PubMed search in June 2022 (using the search terms zebrafish AND inflammation) revealed a total of 1697 published papers for the years between 2000 and 2022. The increasing interest in the use of zebrafish to investigate inflammatory responses is further illustrated by the knowledge that in 2000 there was only one published paper in this area, whereas in 2021 there were 308 publications (with >70% of the existing papers published in the last five years).

It is often desirable to monitor inflammatory responses over time in animal models as this allows capture of the initiation, peak, and resolution (or lack thereof) of inflammation. Methods have been developed to visualize immune cell migration in rodents in real time using intravital microscopy ( Khandoga *et al*., 2009, Xu, Lei and Liu, 2011, reviewed by De Filippo and Rankin, 2020). However, this approach is not routinely used, requires specialized equipment, is not applicable to all tissues/organs and typically assesses responses over short time frames (several hours). Instead, it is common to include several groups of rodents in each *in vivo* study to investigate inflammatory responses that are activated at different time points, with the accumulation of immune cells typically assessed by performing differential cell counts (e.g. from bronchoalveolar lavage (BAL) or blood samples) or by visualizing the inflammatory response using histology.

**Figure 1.**
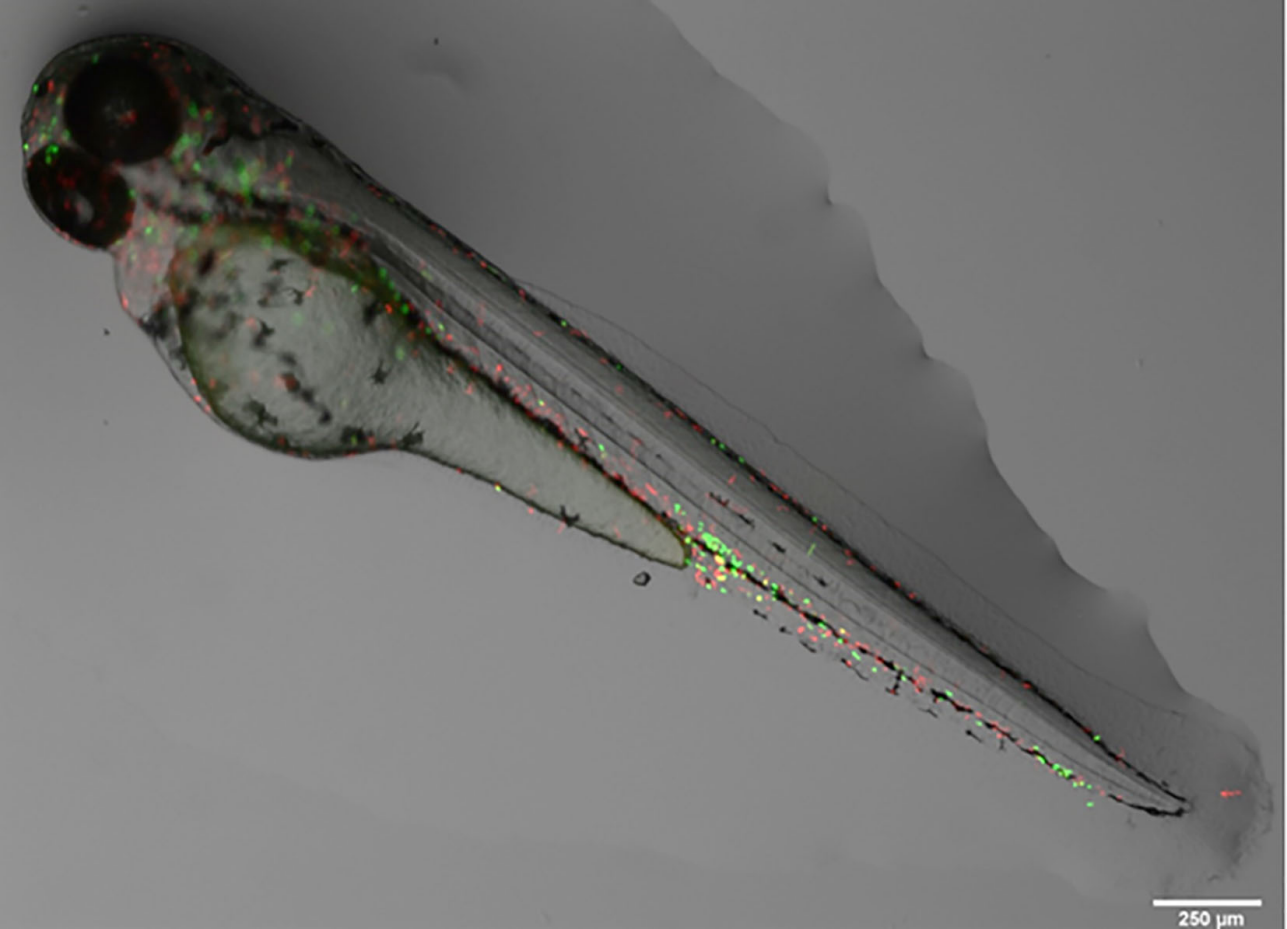
Representative image of a Tg (mpx: EGFP/mpeg:mCherry) larvae (3 dpf) with fluorescent neutrophils (green) and macrophages (red). Image taken by Dr Suzanne Gillies using a fluorescent Leica M205 FCA stereomicroscope. Scale bar is 250 μm.

Histological examination has also been used to visualise inflammatory responses in wild type zebrafish at various life stages (e.g., Yang *et al*., 2014). However, the availability of transgenic zebrafish which express fluorescent proteins in specific immune cell types allows the direct visualization and quantification of immune cell accumulation in specific locations as well as the whole organism ( Figure 1). Importantly, pigmentation starts developing in zebrafish from 1 dpf, and although is not fully developed until 14 dpf ( Rawls *et al*., 2001) this can make it challenging to visualize fluorescent cells as zebrafish age. For this reason, the majority of existing studies typically assess inflammatory responses in early life stages. However, there are several strategies that can be used to improve the transparency of zebrafish larvae, such as the use of (toxic) chemicals to stop the creation of pigment (e.g., 1-phenyl-2-thiourea, PTU) or the use of mutant strains which lack skin pigmentation (e.g., *casper* strains of zebrafish) ( Antinucci and Hindges 2016). To date, the majority of existing research has focused on assessing neutrophil and macrophage responses in zebrafish, using transgenic lines with fluorescent neutrophils (e.g., Tg (mpx: GFP)), fluorescent macrophages (e.g., Tg (mpeg1):GFP) ( Renshaw *et al*., 2006, Ellett *et al*., 2011) or both (e.g., Tg (mpx: GFP/mpeg1:mCherry)) ( Ellett *et al*., 2011). It is most common for images of zebrafish to be captured at specific time points using fluorescent microscopy to visualize and quantify the inflammatory response over time, however imaging can also be performed in real-time. As inflammatory responses can be monitored in the same organism over time this can lead to a reduction in animal use, which is a major benefit of using transgenic zebrafish over rodents. More specifically, studies using (non-protected life stages of) zebrafish commonly investigate inflammatory responses to specific stimuli (e.g., injury, injection of pathogens) at multiple time points (ranging from one to seven, with an average of three) in the same organism, ( Tables 1 and 2). As a comparison, a PubMed search in Oct 2022 (search terms: nanomaterial OR nanoparticle AND lung AND rat or mouse AND inflammation AND neutrophil) revealed that rodent studies used an average of three time points (range of one to six) to quantify pulmonary inflammatory responses to NMs. Therefore, each rodent study investigating inflammatory responses to NMs required the use of multiple groups of animals to gather information at each time point of interest, with separate control groups required for each time point. Zebrafish allow the inflammatory response to be monitored in the same organism over time to maximise the amount of information obtained from each organism, perform a more comprehensive assessment of the dynamics of the inflammatory response (via the use of multiple time points) and reduce the number of treatment groups (and therefore number of animals) required (compared to rodents).

Transgenic zebrafish with fluorescent immune cells have been used to investigate inflammatory responses to various stimuli including, for example, lipopolysaccharide (LPS) (e.g., Yang *et al*., 2014, Zhang *et al*., 2016), neutrophil chemoattractants (e.g., chemokine (CXC) ligand-8 (CXCL-8), N-Formyl-Met-Leu-Phe (fMLF), Leukotriene B _4_ (LTB _4_) ( Elks *et al*. 2011, Bischel *et al.* 2013, de Oliveira *et al*., 2013)), micro-organisms (e.g. Nguyen-Chi *et al.,* 2014, Benard *et al*., 2012, Jim *et al.,* 2016), injury (tail fin wound; (e.g., Renshaw *et al*., 2006, Ellett *et al*., 2011, Hoodless *et al*., 2016, Miskolci *et al*., 2019, Table 1), burns (thermal injury) ( Miskolci *et al*., 2019) and cardiac injury (e.g., Kaveh *et al*., 2020)). In addition, transgenic zebrafish have been used to investigate the contribution of inflammation to disease pathogenesis (e.g. pulmonary disease ( Zhang *et al*., 2016), cardiovascular disease ( Taylor *et al*., 2019, Kaveh *et al*., 2020), intestinal disease ( Brugman, 2016), liver disease ( Shwartz *et al.,* 2019), cancer ( Yang *et al*., 2019, Wrighton *et al.* 2019)), and tissue repair/regeneration ( Renshaw *et al*., 2006, Li *et al*., 2012, Petrie *et al.,* 2014). Transgenic zebrafish have also been used for drug discovery to identify novel anti-inflammatory compounds (e.g., Hoodless *et al*., 2016, Lucas *et al.* 2013, Yang *et al*., 2014, Rahman *et al*., 2019, Xie *et al.,* 2021).

## Nanomaterials and inflammation

Nanomaterials (NMs) are defined as having at least one dimension that is 1–100 nm in diameter ( European Commission, 2011). The properties exhibited by materials at the nanoscale can be drastically different to those observed in larger forms of the same material. This has promoted the incorporation of NMs into a wide variety of consumer products ( Vance *et al*., 2015) with the use of NMs now spanning a very wide range of sectors (e.g., pharmaceuticals, cosmetics, textiles, food, electronics, automotive, construction, agriculture, and pigments/inks). Although the prevalence of NMs in the marketplace continues to increase, there are uncertainties regarding their potential adverse impact on human health. Thus, a thorough assessment of NM toxicity needs to be conducted in parallel to exploring their benefits to ensure that nanotechnology develops in a safe, responsible and sustainable manner. NMs are an extremely diverse group of materials and due to the large number of NMs for which safety needs to be assessed there is a desire to move away from rodent testing to improve alignment of nanotoxicology research with the 3Rs principles ( Burden *et al*., 2017, Johnston *et al*., 2018).

Investigation of whether NMs activate inflammatory responses is routinely used to assess their toxicity both *in vitro* and *in vivo* (reviewed by Johnston *et al*., 2018). *In vitro* studies have used a variety of cell types from different target sites (e.g., immune system, lung, liver, intestine, skin) to assess whether NMs can stimulate cytokine production as an indicator of their potential to activate inflammatory responses. Such studies have shown that, depending on their physico-chemical properties, NMs can enhance the production of neutrophil (e.g., interleukin (IL)-8, growth-regulated oncogene alpha (GROα), macrophage inflammatory protein (MIP)-2) and macrophage (e.g., macrophage chemotactic protein (MCP)-1) chemoattractants as well as other pro-inflammatory cytokines (e.g., tumour necrosis factor (TNF)-α, IL-1β) (e.g., Verdon *et al*., 2021, Zhang and Monteiro-Riviere, 2019, Dankers *et al.,* 2018, Johnston *et al*., 2015, Braakhuis *et al.,* 2016, Wiemann *et al*., 2016, Sahu *et al*., 2014, Kermanizadeh *et al.,* 2013, Farcal *et al.,* 2013, Goncalves *et al.,* 2010, Brown *et al.,* 2004). Investigation of the activation of immune cells *in vitro* (typically macrophages and neutrophils) following exposure to NMs has also been evaluated via investigation of a range of cell responses (e.g., cytotoxicity, cytokine production, activation of respiratory burst, phagocytic function, cell motility, and neutrophil extracellular trap formation (NETosis)) (e.g., Goncalves *et al.,* 2010, Sahu *et al*., 2014, Wiemann *et al*., 2016, Johnston *et al*., 2015, Verdon *et al*., 2021). Whilst cell based, *in vitro* assays often offer a quick, cheap and high-throughput method of assessing the inflammatory effects of NMs, they are not able to accurately reflect the *in vivo* situation as they cannot mimic the complexity of interactions that occur between immune and non-immune cells during inflammatory responses in humans. Furthermore, whilst the production of both pro- and anti-inflammatory mediators can be monitored over time using *in vitro* models, it is challenging to investigate the resolution of inflammatory responses outside an intact organism.

Rodent studies have therefore been used to investigate the dynamics of inflammatory responses that are activated by NMs, and to date the majority of these have focused on pulmonary inflammatory responses. The activation of an inflammatory response (i.e., infiltration of leukocytes into the target site) and failure of that response to resolve *in vivo* is indicative that a NM may be toxic. Studies have consistently demonstrated that NMs (depending on their physico-chemical properties) can activate pro-inflammatory responses that are often characterised by an accumulation of neutrophils (e.g., Brown *et al*., 2001, Poland *et al*., 2008, Ma-Hock *et al*. 2009, Srinivas *et al*., 2012, Murphy *et al*., 2013, Bonner *et al.,* 2013, Kim *et al*., 2016, Landsiedel *et al*. 2014, Gosens *et al*., 2015, Halappanavar *et al.,* 2019). Macrophages also play a key role in the response of organs/tissues (e.g., lungs) to NMs, and the clearance of NMs by resident macrophages is likely to promote an inflammatory response (e.g., Ogawara *et al*., 1999, Sadauskas *et al*., 2007, Semmler-Behnke *et al*., 2008, Geiser *et al*., 2008, Roberts *et al*. 2013, Anderson *et al*., 2015, Gustafson *et al*., 2015).

## Zebrafish and nanotoxicology

### Ecotoxicity

To date, the majority of nanotoxicology studies that have employed zebrafish have focused on investigating the toxicity of NMs to the environment. Such studies have assessed NM hazards via evaluation of the impact of NMs on zebrafish survival, growth, development, reproduction, hatching rate, and teratogenicity following aqueous exposure (e.g., Lee *et al*., 2007, Usenko *et al*., 2008, Asharani *et al*., 2008, Zhu *et al*., 2009, Fent *et al*., 2010, Massarsky *et al*., 2013, Duan *et al*., 2013, Pavagadhi *et al*., 2014, Wehmas *et al*., 2015, Ganesan *et al*., 2016, Pecoraro *et al*., 2017, Vranic *et al*., 2019, Aksakal and Ciltas, 2019). It is most common for such studies to use wild type zebrafish.

Transgenic zebrafish have been used to a more limited extent to assess the potential detrimental impact of NMs on human health via investigation of responses such as inflammation (e.g., using the Tg (mpx: EGFP) ^i114^ strain which expresses fluorescent neutrophils), cardiovascular toxicity (e.g., using the Tg (fli-1:EGFP) strain which expresses EGFP-labelled vascular endothelial markers, fli-1), and neural toxicity (e.g., using the Tg (HuC-GFP) strain that expresses GFP in the central nervous system) (e.g. Bar-Ilan *et al*., 2012, Duan *et al*., 2016, Gao *et al*., 2016, Vranic *et al*., 2019, Gu *et al*., 2021, Gillies *et al.,* 2022).

### Inflammation

Inflammatory responses have been evaluated in wild type zebrafish following aqueous exposure through histological examination of the gills ( Filho *et al*., 2014) and skin ( McLeish *et al.* 2010). In addition, Brun *et al*., (2018) demonstrated that the expression of pro-inflammatory genes and infiltration of neutrophils increased in the skin and intestine of transgenic zebrafish embryos (with fluorescent TNFα, IL-1β, neutrophils or macrophages) following aqueous exposure to copper and polystyrene NMs. A small number of published studies have investigated inflammatory responses to NMs following microinjection into various body sites of transgenic zebrafish larvae with fluorescent neutrophils (e.g., Sharif *et al*., 2012, Zhang *et al*., 2016, Brun *et al*., 2018, Duan *et al*., 2016, 2018, Gillies *et al*., 2022). Only one study was identified in the literature that has investigated inflammatory responses to NMs in (injured) transgenic zebrafish following aqueous exposure ( Gillies *et al*., 2022). Therefore, whilst transgenic zebrafish have been used to assess NM toxicity, these studies have assessed a very limited number of NMs, administration routes, target sites, time points, zebrafish strains and life stages. There is an opportunity now to apply transgenic zebrafish to a far greater extent for assessing the potential adverse impacts of NMs on human health. We therefore propose that the more widespread screening of NM toxicity via investigation of inflammatory responses in transgenic zebrafish could make nanotoxicology testing more ethical, faster, cheaper and potentially more predictive.

### Aims

The use of transgenic zebrafish in nanotoxicology studies to investigate impacts of NMs on human health is in its infancy, however results from existing studies are very promising. For example, as part of an NC3Rs funded project, we assessed the suitability of using transgenic zebrafish to investigate whether NMs could stimulate an inflammatory response in a novel approach to assess their toxicity ( Gillies *et al*., 2022). We selected silver (Ag) NMs, and zinc oxide (ZnO) NMs as *in vivo* rodent data on their capacity to elicit a pulmonary inflammatory response already existed (e.g., Gosens *et al*., 2015, Li *et al*., 1996, Landsiedel *et al*., 2014). Furthermore, there is extensive evidence that these NMs stimulate cytokine production by various cells types *in vitro* (e.g. Gaiser *et al.,*2013, Kermanizadeh *et al.,* 2013, Farcal *et al.,* 2015, Johnston *et al*., 2015, Verdon *et al.,* 2021). Human exposure to these NMs is also expected due to the use of these NMs in a range of consumer products. We investigated the dynamics of the neutrophil responses that were activated by NMs following i) aqueous exposure of injured transgenic zebrafish larvae (with fluorescent neutrophils) or ii) microinjection into the otic vesicle of transgenic zebrafish larvae ( Gillies *et al*., 2022). Only non-protected life stages of zebrafish were used in our studies to support the implementation of the 3Rs principles. We demonstrated that transgenic zebrafish can be effectively employed to assess the inflammatory effects of NMs ( Gillies *et al*., 2022) and would therefore encourage the more widespread use of the model by the nanotoxicology community.

As there is increasing interest in the exploitation of zebrafish to assess inflammatory responses in nanotoxicology and other disciplines, we believe it is timely to present recommendations to new users of the model. There is no standard protocol available to assess inflammatory responses to NMs (or other substances/pathogens) using transgenic zebrafish and within the existing literature the methodology used in different studies to investigate inflammatory responses in transgenic zebrafish is varied (e.g. with respect to the zebrafish strain, life stage, administration route, sample size and time points used as well as the approach used to quantify the inflammatory response) which can make it challenging for new users of the model to design their experiments. Here we therefore provide guidance to new users on how transgenic zebrafish can be used to investigate inflammatory responses to NMs. We will also identify gaps in knowledge to help prioritise future research activities. Importantly, the themes we discuss are not restricted to NMs and are relevant to other substances and pathogens. Furthermore, we focus on assessment of impacts relaying more to human health but many of the topics covered are also relevant to ecotoxicology studies.

## Selection of exposure route/target site

It is important to consider how zebrafish should be exposed to NMs if designing work to infer for effects for exposure routes that are relevant to humans. Assessment of NM toxicity following pulmonary exposure (e.g., inhalation, intratracheal instillation) has dominated existing *in vivo* rodent studies ( Stone *et al*., 2016). Relatively fewer rodent studies have exposed rodents to NMs orally, via the skin or intravenous injection ( Stone *et al*., 2016); however, exposure of humans to NMs via these routes is expected in occupational, environmental, and consumer settings.

**Figure 2.**
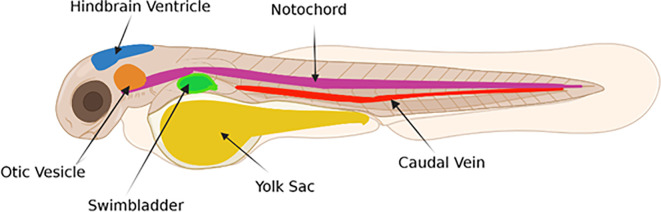
Possible sites of NM microinjection in zebrafish larvae. Figure created in BioRender.com.

The most convenient and popular way of exposing zebrafish to substances (e.g., NMs) is via water ( Sipes *et al*., 2011), but this may not always be the most appropriate route when considering relevance for human health related research. Whilst microinjection is more technically challenging and requires access to specialised equipment, it allows for the administration of NMs into specific sites and subsequent investigation of inflammatory responses locally at the injection site, as well as systemically. Indeed, inflammatory responses to a range of different stimuli (such as LPS, neutrophil chemoattractants and pathogens (e.g., bacteria, fungi)) have been investigated following their microinjection into several sites in zebrafish embryos/larvae including the otic vesicle, swimbladder, hindbrain ventricle, notochord, and caudal vein (e.g., Gratacap *et al*., 2014, Nguyen-Chi *et al*., 2014, Benard *et al*., 2012, Jim *et al*., 2016, Harvie and Huttenlocher 2015, de Oliveira *et al*., 2013) ( Figure 2). Recommendations are provided below regarding how zebrafish can be exposed to NMs when assessing responses following pulmonary, oral, and dermal exposure as well as intravenous injection.

## ‘Pulmonary’ exposure

We have outlined below how zebrafish may be exposed to NMs via water or microinjection to mimic pulmonary (respiratory) exposure.

### Aqueous exposure

Interestingly, zebrafish have been used to assess the pathogenesis of pulmonary diseases, despite their lack of a mammalian-like respiratory system ( Renshaw *et al.* 2007, López Hernández *et al*., 2015, Progatzky *et al*., 2016, Zhang *et al*., 2016). Fish respire via gills, and these tissues perform an equivalent gas-exchange function to mammalian lungs ( Progatzky *et al*., 2016), as well as playing a key role in other critical functions such as ion and pH regulation and the excretion of nitrogeneous waste ( Evans *et al*., 2005, Dymowska *et al*., 2012). The zebrafish gill is the primary site of gas exchange and has a complex vasculature that is covered by an epithelium which acts as a barrier between the blood and external environment ( Evans *et al*., 2005). Within the structure of the gills there are several different cell types including epithelial cells, ionocytes (which control ion transport), inflammatory cells (e.g., lymphocytes and neutrophils), mucus-producing (goblet) cells, and skeletal muscle cells. Unidirectional flow of water over the gills (as opposed to tidal flow of air in lungs of mammals), production of mucus, and coughing during ventilatory movements combine to enable fish to dislodge and remove materials (e.g., particulates, including NMs) from gill surfaces.

There is evidence that NMs can accumulate in the gills of fish following aqueous exposure ( Johnston *et al*., 2010, Zhao *et al*., 2011, Bar-Ilan *et al*., 2013, Bacchetta *et al.,* 2016, Skjolding *et al*., 2017). For example, it has been shown that the gills of zebrafish are the primary site of copper oxide (CuO) NM accumulation and toxicity following aqueous exposure ( Griffitt *et al*., 2007). Furthermore, Tang *et al*., (2019) demonstrated that TiO _2_ NMs could cause oxidative damage to the adult zebrafish gill following aqueous exposure. Interestingly, Filho *et al*. (2014) showed (using histological examination) that carbon nanotubes (CNTs) stimulated an inflammatory response in the adult zebrafish gill following aqueous exposure. Given the above, inflammatory responses in the gills of zebrafish in response to NM exposure via water could provide a potential surrogate for assessing pulmonary inflammatory responses in rodents. However, zebrafish gills do not start to develop until 3 dpf, and they are not fully developed and functional until >14 dpf ( Rombough, 2002). Therefore, protected life stages of zebrafish would likely have to be used for assessing NM mediated inflammatory responses at this target site, with the associated ethical implications and animal licencing requirements. Using protected life stages of zebrafish, however, may still have 3Rs benefits as it allows for a reduction in the use of mammals in toxicology testing and it may be possible to monitor responses in the same organism over time due to the transparency of early life stages of zebrafish, which will reduce the number of animals (fish) used in each experiment. However, this will depend on whether the increased pigmentation of zebrafish which increases as they age obscures the visualization of fluorescent immune cells in transgenic zebrafish (see above).

The skin is the main site of gas exchange in zebrafish embryo/larvae until the gills are developed, and it has been suggested that the skin epithelium has a similar function to the alveolar epithelium in humans ( Rong *et al*., 2021). The skin epithelia could therefore have potential for use to assess the respiratory toxicity of NMs following aqueous exposure in non-protected life stages of zebrafish ( Schwerte, 2010).

Aqueous exposure of zebrafish to NMs is not technically challenging (e.g., Gillies *et al*., 2022), does not require specialised equipment and has the added benefit that repeated exposures can be performed with the same animals. Thus, transgenic zebrafish with fluorescent immune cells could potentially be used to investigate inflammatory responses to NMs at the gills or skin as an alternative to assessing inflammatory responses following pulmonary exposure in rodents.

### Microinjection

The swim bladder is a gas filled sac which regulates buoyancy in zebrafish and has anatomical similarities in its cellular make up to the alveoli of the human lung ( Finney *et al*., 2006). Furthermore, the swim bladder shares a common evolutionary origin with the mammalian lung ( Zheng *et al*., 2011). The swim bladder has three distinct layers of tissue; a single layer of epithelial cells covering a mesenchymal layer which is then surrounded by an outer mesothelial layer ( Yue *et al.,* 2015). The epithelium is lined with a thin fluid (mucosal) layer containing surfactants (as in the mammalian lung) ( Prem *et al.,* 2000). Zhang *et al*., (2016) observed that silica NMs, LPS, Polyinosinic:polycytidylic acid and neutrophil chemoattractants (IL-8 and MIP-2) stimulated an infiltration of neutrophils into the swim bladder of transgenic zebrafish larvae following microinjection. No other studies were identified in the literature that have injected NMs into the swim bladder of zebrafish to investigate their toxicity. Investigation of inflammatory responses in the swim bladder of transgenic zebrafish larvae (with fluorescent immune cells) has been used in previous studies to investigate lung injury ( Zhang *et al*., 2016, Lee *et al*., 2019), as well as host-pathogen interactions (e.g. the fungal pathogen *Candida albicans)* ( Gratacap *et al*., 2014). Thus, microinjection of NMs into the swim bladder could also be an appropriate method to investigate pulmonary responses to NMs. Gratacap *et al*., (2014) provide guidance on how to perform swim bladder microinjections for fungal pathogens. However, the swim bladder is not fully developed until 4–5 dpf and so investigation of NM mediated inflammatory responses in this location may require the use of protected life stages of zebrafish (depending on how long inflammatory responses were monitored for) and thus there may be more ethical implications of using this exposure route and again permission will be required from responsible authorities to perform this type of research.

## Oral exposure

The toxicity of NMs following oral exposure has been investigated in several rodent studies (e.g., de Jong *et al*., 2019, Park *et al*., 2010, Heo *et al.*, 2020) and thus it is appropriate to consider how zebrafish can be used to investigate the fate and toxicity of ingested NMs. Exposure of fish to NMs via water can lead to NM accumulation in the gastrointestinal tract (GIT) via drinking (e.g., Lin *et al*., 2014, Zhao *et al*., 2011, van Pomeron *et al*., 2017). Alternatively, NMs can be incorporated into the diet of fish (e.g., Geffroy *et al*., 2012, Merrifield *et al*., 2013). Indeed, localisation of NMs in the intestine has been observed in zebrafish exposed to NMs via the diet ( Skjolding *et al*., 2017). Ingestion of food containing Ag and Cu NMs by adult zebrafish for 14 days has been observed to disrupt the gut microflora ( Merrifield *et al.,* 2013). Furthermore, Brun *et al*. (2018) observed that intestinal tissue pro-inflammatory cytokine expression was increased, and that there was an increase in macrophage and neutrophil accumulation in the intestine following aqueous exposure to Cu NMs. Zebrafish have also been successfully used to investigate the role of inflammation in intestinal diseases (reviewed by Brugman, 2016). The gastro-intestinal tract opens after 3 dpf, however zebrafish do not feed independently until ~5 dpf, up until which time they are reliant on a yolk sac reserve. Consequently, protected life stages of zebrafish are required to investigate the toxicity of ingested NMs. Given the absence of studies which have investigated inflammatory responses to NMs in the intestine following ingestion this would seem to be an area of research that should be prioritised for the application of transgenic zebrafish in the future.

## Dermal exposure

The majority of existing studies have used *in vitro* approaches to assess the dermal toxicity of NMs (e.g., Monteiro-Riviere *et al*. 2005, Rouse *et al*. 2008, Sayes *et al*. 2006, Shvedova *et al*., 2003, Zhang *et al*., 2008, Connolly *et al*., 2019, reviewed by Crosera *et al.,* 2009) and far fewer studies have investigated dermal toxicity of NMs *in vivo* (e.g. Samberg *et al*., 2010). Cytokine production by *in vitro* skin models of varied complexity (e.g., monocultures of skin cells (e.g., keratinocytes), complex 3D models of the skin (e.g., EpiDerm, and human/animal tissue) can be used to investigate whether NMs activate an inflammatory response. Several *in chemico* and *in vitro* OECD test guidelines (that recommend the use of more advanced *in vitro* models) are available that can be used to screen the dermal toxicity of substances (e.g. via assessment of irritation and sensitisation) (e.g. OECD Test Nos 432, 439, 442C, 442E). Indeed, the dermal toxicity of NMs has already been screened using OECD methods (e.g., Park *et al*., 2011, Bezerra *et al*., 2021). Complex, physiologically relevant 3D skin models (with multiple cell layers) have also been used to assess NM toxicity *in vitro* as an alternative to animal testing and most commonly to assess skin irritation (loss of cell viability) (e.g., Park *et al*., 2011, Choi *et al*., 2014, Kim *et al*., 2016, Miyani and Hughes 2017, Franková *et al*., 2018, reviewed by Sanches *et al*., 2020). It is also common for researchers to assess the response of (a single layer of) keratinocyte cell lines when investigating the dermal toxicity of NMs *in vitro.* For example, cytokine production by keratinocytes has been used to screen the dermal toxicity of NMs *in vitro* ( Samberg *et al.,* 2010, Saathoff *et al.,* 2011, Jeong *et al.,* 2013, Murray *et al.,* 2013, Galandáková *et al.,* 2016). However, there is evidence that more simple, less physiologically relevant models are more sensitive to NM toxicity ( Chen *et al*., 2019). Whilst 3D *in vitro* models of the skin are available and have been used as an alternative to animal (rodent) testing, they lack the ability to assess the migration and accumulation of immune cells in the skin following NM exposure. Assessment of such responses is desirable to assess the contribution of inflammation to the dermal toxicity of NMs. Non protected life stages of zebrafish may therefore offer an alternative model to assess the dermal toxicity of NMs *in vivo* and more specifically to investigate the activation of inflammatory responses within the skin. Indeed, there is evidence that NMs accumulate in the skin of zebrafish following aqueous exposure (e.g., Zhao *et al*., 2011, van Pomeron *et al.,* 2017), and that NMs can exhibit toxicity to the skin of zebrafish. For example, Brun *et al*., (2018) found that CuO NMs increased the expression of the pro-inflammatory cytokine IL-1β and the accumulation of immune cells (such as macrophages and neutrophils) in the skin of transgenic zebrafish embryos, following aqueous exposure. In addition, McLeish *et al*., (2010) demonstrated (using histology) that NMs caused skin damage in zebrafish larvae, and induced leukocyte infiltration into the epidermis following aqueous exposure. More recently, exposure of zebrafish to Cu and Ag NMs has been shown to cause oxidative stress in various types of ionocytes which are cells that are present in the skin and responsible for mediating ion exchange in zebrafish embryo/larvae (Takesono *et al*., manuscript in preparation).

Zebrafish embryos are protected by a chorion up to 3 dpf that acts as a barrier and limits the interaction of NMs (and other substances) with the embryo ( Brun *et al*., 2018, van Pomeron *et al.,* 2017, Fent *et al*., 2010). This has major implications when considering the life stage that is used to assess the dermal impacts of NMs on zebrafish, and it may be preferable to use zebrafish embryos that have hatched from the chorion ( Brun *et al*., 2018). Alternatively, embryos can be dechorionated manually or by using chemicals/enzymes to allow skin exposure at earlier time points, although this can be time consuming. Following hatching, the main route of exposure in non-protected life stages will be via the dermal route, as the gills are not fully developed and the embryo cannot independently feed at this time ( Sipes *et al*., 2011). Due to the limited number of studies which have investigated inflammatory responses to NMs in the skin of transgenic zebrafish, this is a knowledge gap that needs researching.

When assessing inflammatory responses in zebrafish embryo/larvae following dermal exposure it may be of interest to investigate the responses of neuromasts. Neuromasts are mechanosensory cells that comprise the lateral line system but are also found more widely across the body surfaces of zebrafish ( Froehlicher *et al*., 2009). It has been suggested that investigation of the impact of chemicals on neuromast development and function can be used to assess toxicity (reviewed by Froehlicher *et al*., 2009). Previously, neuromast cell death has been assessed to investigate the toxicity of metals, such as copper, and pharmaceuticals, (e.g., Mourabit *et al.*, 2019). In addition, McNeil *et al.,* (2014) demonstrated that exposure of zebrafish embryos to Cu NMs reduced the number of functional lateral line neuromasts which affected fish behaviour. As only a few studies have assessed the toxicity of NMs to neuromasts this should be explored further in the future to address knowledge gaps.

## Intravenous exposure

Intravenous injection provides a way of directly introducing NMs into blood at a specific dose and can be used to identify the implications of NMs on health if they become systemically available following exposure. Exposure of rodents via intravenous injection is commonly used to investigate the fate and toxicity of NMs that may be used in a clinical setting (e.g., nanomedicines) as they may be administered via this route. Several rodent studies have investigated NM biodistribution and toxicity following intravenous injection (e.g., Semmler-Behnke *et al.,* 2008, Fujihara *et al.,* 2015, Disdier *et al.,* 2015). It is therefore prudent to reflect on how to administer NMs to zebrafish to mimic intravenous injection. When investigating host-pathogen interactions in zebrafish larvae, pathogens have frequently been injected into the caudal vein or Duct of Cuvier (a circulation channel located on the yolk sac, and responsible for connecting the heart to the trunk vasculature) to cause systemic blood infections ( Benard *et al*., 2012, Harvie and Huttenlocher, 2015). In addition, the cardiotoxicity of silica NMs following their injection into the Duct of Cuvier has been investigated previously, with cardiac inflammation (neutrophil accumulation) observed following NM exposure ( Duan *et al*., 2016). As investigation of whether NMs can activate systemic inflammatory responses following intravenous injection into non-protected life stages of transgenic zebrafish larvae has been neglected to date it is suggested that this knowledge gap is addressed in future studies.

The yolk sac contains a supply of nutrients required by the developing zebrafish until exogenous feeding beings at 5 dpf ( Quinlivan and Farber 2017). Microinjection of pathogens and substances into the yolk sac of zebrafish has been used as a systemic infection/exposure model ( Benard *et al*., 2012, Yang *et al*., 2014, Jim *et al*., 2016, Xie *et al*., 2021). The distribution of fluorescent polystyrene NMs following microinjection into the yolk sac has also been investigated in zebrafish embryos ( Zhang *et al*., 2020); however no other published studies were identified in which the fate or toxicity of NMs following injection into the yolk sac were investigated. Administration of NMs via the yolk sac could be an effective way to introduce NMs such that they become systemically available and therefore this route of exposure could be used as a surrogate to intravenous injection, but more studies specifically focusing on NMs are required to substantiate this.

## Other exposure routes

Several routes of administration have been discussed above to outline how zebrafish may be exposed to NMs to mimic pulmonary, dermal and oral exposure and intravenous injection. Other administration routes can also be used that do not necessarily mimic a specific route of human exposure but can nevertheless be used to screen the inflammogenicity of NMs (and other substances/pathogens). Importantly, the use of a wider range of exposure routes provides an opportunity to make testing more ethical by promoting the use of non-protected life stages of zebrafish.

### Otic vesicle

The otic vesicle is the ear of the zebrafish and is a closed hollow cavity that is usually devoid of immune cells ( Haddon and Lewis 1996, Harvie and Huttenlocher, 2015). An infiltration of leukocytes (e.g., neutrophils, macrophages) into the otic vesicle has been observed following microinjection of different stimuli such as CXCL-8, fMLF, LTB _4_, LPS, and bacteria ( Harvie and Huttenlocher 2015, Benard *et al.,* 2012, de Oliveira *et al.,* 2013). In our work, we have demonstrated that injection of Ag NMs into the otic vesicle of early life stages of zebrafish (3 dpf) stimulated an infiltration of neutrophils to this site ( Gillies *et al*., 2022). However no other studies could be identified that have investigated the toxicity of NMs following their microinjection into the otic vesicle. Benard *et al*., (2012) have provided guidance on how to inject pathogens into the otic vesicle and monitor inflammatory responses, which can be adapted for NMs. When performing a microinjection into any site in zebrafish embryos/larvae it is recommended that a tracer molecule (such as dextran) is used to confirm the successful injection of the target site. Based on our own studies ( Gillies *et al*., 2022) we suggest that injection into the otic vesicle of transgenic zebrafish offers an effective way to assess for inflammatory responses to NMs.

### Hindbrain ventricle

Brain ventricles are cavities in the brain of zebrafish that contain cerebrospinal fluid ( Gutzman and Sive 2009). Inflammatory responses to range of pathogens (e.g., bacteria ( Benard *et al*., 2012, Rocker *et al*., 2015 Jim *et al*., 2016, Du *et al.,* 2017, Mazon-Moya *et al*., 2017), and fungi ( Brothers and Wheeler, 2012, Torraca *et al*., 2015, Voelz *et al*., 2015)) have been investigated in transgenic zebrafish (with fluorescent immune cells) following their microinjection into the hindbrain ventricle. The hindbrain ventricle is initially devoid of leukocytes, but over time (from 35 hpf) there is a progressive increase in the number of resident immune cells (such as macrophages (microglia)) ( Herbomel *et al*., 2001). This has sometimes led to investigators selecting other exposure sites which are devoid of leukocytes when investigating inflammatory responses to different stimuli ( Harvie and Huttenlocher, 2015) as the quantification of the inflammatory response is easier to perform in leukocyte-free regions. However, macrophages are resident in the organs (e.g., lungs) of rodents and humans, and thus it may be more physiologically relevant to conduct studies in zebrafish where there are resident tissue macrophages. In our searches of the literature, no studies were identified that have investigated NM mediated inflammatory responses following their microinjection into the hindbrain ventricle of zebrafish so this exposure site should be considered in future studies and the use of non-protected life stages prioritised to promote alignment with the 3Rs principles. Guidance is available on how to perform microinjections into the hindbrain ventricle for pathogens, which could be modified for application to NMs ( Benard *et al.,* 2012, Brothers and Wheeler, 2012).

### Tail fin injury model

Transection of the tail fin of non-protected life stages of zebrafish (typically at 3 dpf) causes a wound that stimulates a rapid and robust inflammatory response at the injury site. The inflammatory response to tail fin injury was first characterised by Renshaw *et al*. (2006), who investigated neutrophil responses to injury over time. Whilst the tail fin injury model has been most commonly used to investigate tissue repair and regeneration *in vivo,* the model can also be used for a variety of other purposes. For example, the tail fin injury model has been used to investigate the efficacy of anti-inflammatory drugs that promote the resolution of neutrophilic inflammation (e.g., Hoodless *et al*., 2016, Lucas *et al*., 2013, Rahman *et al.,* 2019) and to investigate inflammatory responses to chemicals and pro-inflammatory stimuli, such as lipopolysaccharide (LPS) ( Cordero-Maldonado *et al*., 2013).

For the first time, we investigated whether aqueous exposure of larval transgenic zebrafish (with fluorescent immune cells) to NMs could enhance the inflammatory response that was activated following a tail fin injury ( Gillies *et al*., 2022). We found that the neutrophil response at the injury site in zebrafish was enhanced and took longer to resolve following aqueous exposure to Ag and ZnO NMs relative to the untreated (injured) controls ( Gillies *et al*., 2022). More recently, we have also investigated neutrophil and macrophage responses to ultrafine carbon black (ufCB) using the tail fin injury model. ufCB is a carbon particle (14 nm diameter) that is commonly used as a surrogate to represent particulate air pollution (PM _10_) in toxicology experiments. PM _10_ is known to cause a spectrum of adverse health effects and inhalation of ultrafine particles (diameter of <100 nm) in PM _10_ are likely associated with the activation of inflammation and oxidative stress in the lungs and other sites (reviewed by Johnston *et al*., 2018). There is evidence from rodent and *in vitro* studies that ufCB can stimulate a pulmonary inflammatory response (e.g., Li *et al*., 1996). Thus, we investigated whether the pro-inflammatory effects of ufCB could be assessed in transgenic zebrafish. We observed that the inflammatory response activated in injured zebrafish is enhanced following exposure to ufCB, as indicated by the increased number of neutrophils and macrophages which accumulate in the injury site compared to the untreated injured control (data not shown). This finding and evidence presented in Gillies *et al*. (2022) therefore demonstrate the utility of the tail fin injury model in investigating NM mediated inflammatory responses.

The tail fin injury is easy to perform and injured zebrafish can be exposed to NMs (or other substances/pathogens) via water to investigate the dynamics of the inflammatory response that is activated. The tail fin injury zebrafish model therefore offers an approach that allows assessment of whether NMs activate an inflammatory response in a relatively fast, efficient way and is not as technically challenging as performing microinjections, enabling it to be more readily widely adopted.

Guidance is available on how to perform a tail fin injury (e.g., Lisse *et al*., 2015), however we have observed that there are differences in the magnitude and the duration of the inflammatory response that is activated in the injury site of zebrafish in published studies ( Table 1). There are several factors which may influence why different studies show different results including: the severity of the injury, the region used to quantify the inflammatory response, the strain of zebrafish used, the time points investigated and sample size used ( Table 1). It is therefore prudent to reflect on what aspects of the experimental design may influence the experimental outcome for this model. In this section we focus on the importance of the severity of the injury and region where the responding immune cells are assessed, with other factors considered later.

**Table 1.** Summary of the experimental design and findings of published tail fin injury studies investigating neutrophil responses in zebrafish. Studies are presented in alphabetical order. The number of larvae included in each group is provided, as well as information on how many independent experiments were included, when available. The mean neutrophil count is provided for each time point, and the neutrophil counts are presented from the shortest to longest time point investigated. The size of the region that was used to perform the neutrophil counts is provided when stipulated in the publication. The numbers of neutrophils accumulating in the injury site are not typically stated in the text of research papers and so the number of neutrophils was often extrapolated from the graphs presented and therefore may not be fully accurate.

Reference	Strain	Injury details	Number of larvae/group (n)	Larvae age at injury (dpf)	Neutrophil count following tail injury (average/fish)	Hours post-injury when response was quantified	Size of Region counted
Boecke *et al*., 2012	Tg (PU1-Gal4-UAS-RFP;MPX-GFP) *Fluorescent leukocytes*	Laser	20	2	4	1.5	Dashed line on images in the publication indicated the area in which leukocytes were counted
van den Bos *et al*., 2020	Tg (mpx: GFP/mpeg1:mCherry-F) *Fluorescent macrophages and neutrophils*	1 mm sapphire blade	43 – 47 (3 independent experiments, n>10 per experiment)	3	19	4	Not reported
Buchan *et al*., 2019	Tg (lyz: MPO-mEmerald; lyz:nfsBmCherry) *Fluorescent neutrophils, and fluorescent MPO*	Scalpel	n = 45 (3 independent experiments)	3	12, 10	3, 6	Dashed line on images in the publication indicated the area in which leukocytes were counted
Cvejic *et al*., 2008	*Immunostaining & Tg (lyz: EGFP)* *Fluorescent neutrophils*	Sharpened tungsten needle	50	3	9 *time lapse imaging also performed	3	Not reported
Deng *et al*., 2011	Tg (mpx:mCherry)	Wounded in the ventral tailfin using a 25 gauge needle	30	3	12	2	Not reported
Gillies *et al*., (2022)	Tg (mpx: EGFP) ^i114^ *Fluorescent neutrophils*	Scalpel	12 per treatment per experiment (≥3 independent experiments)	3	1, 9, 10, 8, 6, 2	0, 4, 6, 8, 24, 48	250 μm from the transected tail fin edge
Hoodless *et al*., 2016	Tg (mpx:eGFPi ^114^) *Fluorescent neutrophils*	Scalpel	≥40 fish/group (3 independent experiments)	3	5, 13, 12, 11, 11	0, 4, 8, 12, 24	0.5 mm length from the tip of the body of the fish
de Oliveira *et al*., 2013	Tg (mpx:eGFPi ^114^) *Fluorescent neutrophils*	Scalpel	30 (3 independent experiments)	3	5, 8, 10, 13	1, 2, 4, 6	Not reported
Lam *et al*., 2013	Not clearly reported: ( *Tg* ( *mpx:dendra2*) or *Tg* ( *mpx:mCherry*) Fluorescent neutrophils	33 gauge needle	Not reported	3	6, 7	1, 4	Not reported
Li *et al*., 2012	Tg (coro1a *:*eGFP *;*lyz *:* Dsred *)* *Fluorescent macrophages and neutrophils*	Not reported	≥15 13 (real time imaging)	3	Neutrophils: 0, 9, 7, 4, 2, 1, 1, 0, 0, 0	0, 2, 6, 12, 24, 36, 48, 60, 72 and 96 h post injury Time lapse imaging also performed with images taken every 3 minutes for 48h	Not reported
Lucas *et al*., 2013	Tg (mpx:eGFPi ^114^) *Fluorescent neutrophils*	Scalpel	n>23 (5 independent experiments)	3	5, 17, 15	0, 4, 24	Area indicated by a line on representative images presented in the publication
Miskolci *et al*., 2019	( *Tg (tnf: GFP) x Tg (lysC:BFP/mpeg1:mCherry-CAAX)*) *Fluorescent macrophages, neutrophils and TNFα*	Scalpel Thermal Injury (burn)	28–44	3	Scalpel: 5, 6, 3, 4, 6, 7 Burn: 10, 20, 8, 12, 11, 9	2, 6, 24, 48, 72, 96	Cell counted in the caudal fin tissue distal to the caudal artery/vein loop
Powell *et al*., 2017	Wild Type zebrafish *neutrophils imaged using Sudan black staining Tg ( *mpx*: Dendra) *Fluorescent neutrophils*	Scalpel	16 25	3 3	7, 9, 6 7, 3	1, 3, 6 3, 6 *time lapse imaging also performed	Not reported
Ren *et al*., 2015a	*Tg (aanat2:EGFP);(lyz: DsRed)* *Fluorescent neutrophils*	Scalpel	Not reported	3	11, 20, 38, 19, 15, 15	0.5, 3,10, 20, 30, 40	Not reported
Ren *et al*., 2015b	Tg (lyz: EGFP) *Fluorescent neutrophils*	Scalpel	Not reported	3	18, 28, 19, 18	3,6, 24, 36	250 μm from the wound ending
Renshaw *et al*., 2006	Tg (mpx:eGFPi ^114^) *Fluorescent neutrophils*	Scalpel	15 (from 5 independent experiments)	4	1, 4, 9, 10, 8, 5 *time lapse imaging also performed	0,1, 2, 4, 8, 24	Region of interest was defined corresponding to the area of inflammation quantitated
Walters *et al*., 2010	Tg (MPO:GFP) *Fluorescent neutrophils*	Wounded in the ventral tailfin using a 25 gauge needle	20-25 (3 independent experiments)	3	0, 3.5, 3.5, 2.5, 2.5, 0.5, 0	0, 1, 3, 6, 8, 12, 24	Not reported
Yang *et al*., 2018	Tg (corola: eGFP) *Fluorescent neutrophils*	Scalpel	15 (3 independent experiments)	3	17	6	Not reported
Zhou *et al*., 2020	*Tg (coro1a*: *GFP*; *lyz*: *Dsred*) *Fluorescent macrophages and neutrophils*	Not Reported	Sample size not reported (although states 3 independent experiments performed)	3	43	4	Not reported

**Figure 3.**
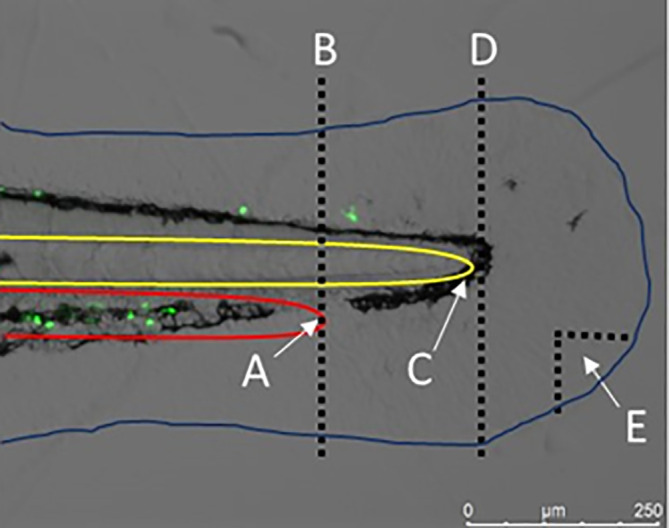
Severity of injury to the zebrafish tail fin. The image shows the tail fin of a 3 dpf Tg (mpx: EFGP) ^114^ larvae (with fluorescent (green) neutrophils) prior to tail fin transection. The edge of the circulatory loop (red) (A), where the caudal artery becomes the caudal vein, can be used as a marker to perform a severe tail fin injury (B). The tip of the notochord (yellow) (C) can be used as a marker to perform a moderate tail fin injury (D). A small nick that is removed from the tail fin can induce a minor injury. The blue line is used to indicate the outline of the zebrafish larvae. Image obtained on a Leica M205 FCA fluorescent stereomicroscope using a Leica DFC7000T digital colour camera, at 160x magnification. Scale bar 250 μm. Image taken by Dr Suzanne Gillies.

The severity of the injury that is inflicted to the tail fin can markedly influence the inflammatory response that is activated ( Elks *et al*., 2011). We have observed that studies do not consistently report on the severity of the injury used, which is an important omission from the methodology. The blood vessels that make up the circulatory loop (where the caudal artery loops back into the caudal vein) can be used as a marker for amputating the tail fin to induce a severe injury, whereby the notochord is transected ( Figure 3), as established by Renshaw *et al*. (2006). A less severe, more moderate injury can be induced whereby the tip of the notochord is used as the marker for transection, avoiding damage to the notochord and blood vessels ( Figure 3) ( Barros-Becker *et al*., 2017, Hoodless *et al*., 2016, Li *et al*., 2012, Lucas *et al*., 2013). A minor injury can be inflicted through making a small tissue nick in the tail fin ( Elks *et al*., 2011; Paredes *et al*., 2019; Tauzin *et al*., 2014). We suggest that a moderate injury is employed when investigating NM toxicity in injured zebrafish to ensure the inflammatory response initiated is robust (i.e., can be detected) but avoids damage to critical structures such as the blood vessels or the notochord, as the latter may make quantification of the response more variable and challenging. Interestingly, different approaches have been used to cause the injury (e.g., the use of a scalpel, needle, or laser), with the use of a scalpel the most common approach ( Table 1). There is evidence that burn wounds (thermal injury) can induce a greater inflammatory response than transection using a scalpel ( Miskolci *et al*., 2019).

Whilst some published studies have reported what region at the injury site was used when counting immune cell accumulation, many do not ( Table 1). Differences in the counting region used in different studies may also help explain why there are differences in the numbers of neutrophils which accumulate at the injury site in the published literature ( Table 1). Accordingly, it is recommended that a standard region for counting the immune cell response region is identified, and in the meantime published studies ensure this information is reported.

## Summary of exposure routes

**Figure 4.**
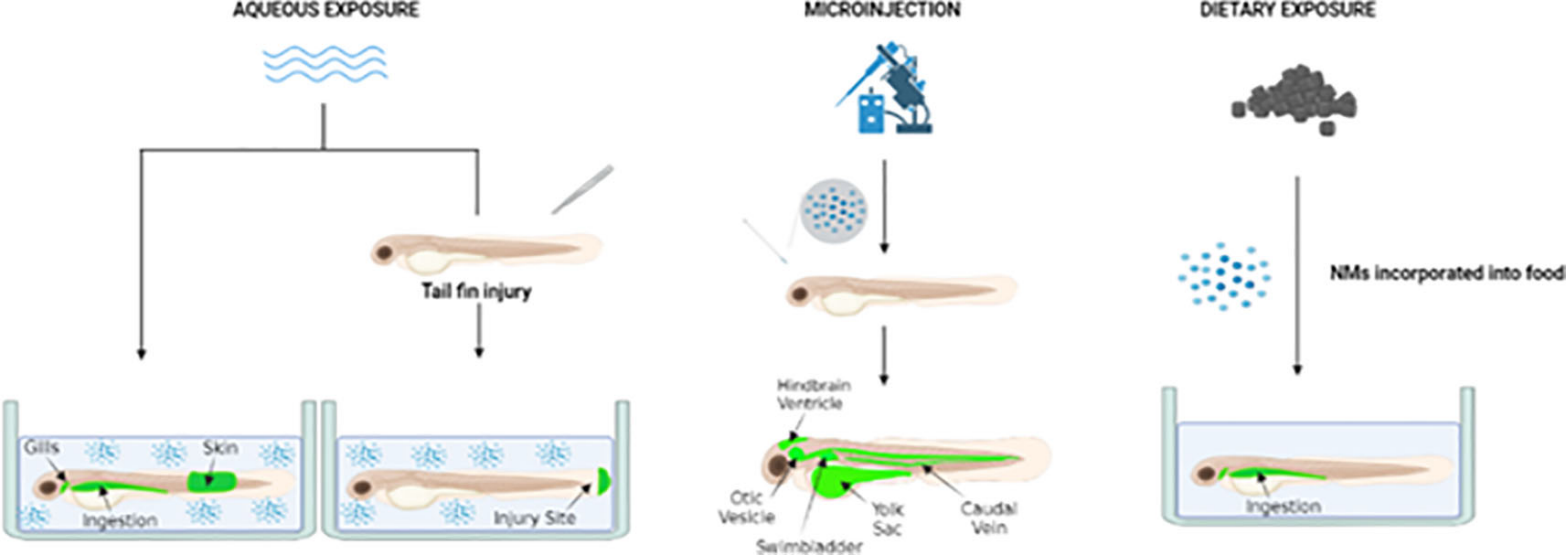
Summary of how zebrafish embryos/larvae may be exposed to NMs. Aqueous exposure of zebrafish to NMs can lead to exposure via the gills, skin or intestine (via drinking or the diet) to investigate responses following respiratory, dermal and oral exposure respectively. Exposure of injured zebrafish to NMs via water can allow for acute inflammatory responses at the injury site (as well as other target sites) to be monitored to screen their toxicity. Microinjection of NMs into the otic vesicle, swim bladder, hindbrain ventricle, caudal vein or yolk sac can allow local and systemic inflammatory responses at the injection site and/or whole body to be investigated. Created using BioRender.com.

We have outlined what routes of administration can be used to expose zebrafish to NMs to provide potential mammalian surrogates for exposure via the lungs, skin and intestine and intravenous injection. We have also discussed what other exposure routes might be considered when assessing whether NMs activate an acute inflammatory response but which are not relevant to a particular exposure route in humans/rodents. A summary of all exposure routes discussed is presented in Figure 4.

The choice of exposure route for nanotoxicology studies that use zebrafish to investigate the impacts of NMs on human health is likely to be dictated by several considerations, including, but not limited to, the following:

1. **The anatomical relevance of the exposure site in zebrafish to the human/rodent exposure route or target site of interest.**
  It is key to consider whether the exposure route selected in zebrafish is relevant to how humans/rodents would be exposed. However, whilst some exposure routes for zebrafish are not directly relevant to a specific route of human/rodent exposure they can still be considered as they can be used to screen NM toxicity and may have ethical benefits (e.g. as they allow for the use of non-protected life stages of zebrafish).
2. **The requirement to assess responses following local or systemic administration, and the need to investigate local and/or systemic responses.**
  Exposure via microinjection allows for targeted administration of substances to specific sites. Alternatively, exposure via water or intravenous injection is often associated with a more widespread distribution of the test substance. The toxicity of NMs may be restricted to the exposure site, or effects may be observed at sites distal to the point of exposure. Rodent studies often focus on investigation of local inflammatory responses that are activated at the exposure site but may also consider whether systemic responses are also stimulated. For example, responses in extra-pulmonary sites such the liver may be investigated following the pulmonary exposure of rodents. Importantly, the site of exposure used for a pathogen/substance in zebrafish will dictate whether the infection/exposure remains localised or can become systemic ( Benard *et al*., 2012). Thus, the requirement to assess local and/or systemic responses should be considered when selecting an exposure route for zebrafish.
3. **The life stage of zebrafish that will be used.**
  Early life stages of zebrafish (<5 dpf) are not protected, and thus it is often desirable to work with early life stages of zebrafish to make testing more ethical. Administration via the otic vesicle, caudal vein/Duct of Cuvier, skin, yolk sac and hindbrain ventricle and exposure of injured fish via water would typically use non-protected life stages of zebrafish, whereas exposure via the gills, swim bladder and ingestion (water or diet) would employ protected life stages ( Figure 4). If protected life stages of zebrafish are used this would require permission from the responsible authorities (e.g., animal licencing and local ethical approval) in the country that the work is performed and their use would not constitute a replacement of animal use. The use of non-protected life stages of zebrafish will restrict what exposure and target sites can be investigated, as systems develop at different rates and times. Whilst there are clear 3Rs benefits of working with non-protected life stages of zebrafish there sometimes also 3Rs benefits of working with protected life stages of zebrafish over rodents. For example, it may be possible to monitor an inflammatory response in the same zebrafish over time in protected life stages leading to reductions in animal use (although this will be dependent on whether the pigmentation of zebrafish obscures visualisation of the target site and whether capser strains are available that lack skin pigmentation).
4. **The need to assess responses following single or repeated exposures**.
  OECD protocols are available to assess toxicity of substances (e.g., NMs) following different exposure routes (such as inhalation, ingestion) in rodents and often require repeated exposure to the substance under investigation. The capacity to perform repeated exposures likely depends on the zebrafish life stage as well as the exposure route that has been selected. For example, whilst repeated exposure to NMs via water is possible, repeated exposures via microinjection are not normally performed and is unlikely to be possible for all microinjection sites, particularly in non-protected life stages.
5. **The requirement to access specialised equipment.**
  Microinjection is technically challenging, time consuming and requires specialized equipment which may not always be available to researchers, and as a consequence exposure of zebrafish larvae to NMs via water is likely to be the preferred route of exposure. However, it has to be accepted that whilst each individual fish may have the same exposure level to the test substance when exposed via water that the uptake of NMs by individual fish may vary, which is a complicating factor for this exposure route. Accordingly, assessing NM fate in zebrafish following exposure via water (as well as other routes) in parallel to evaluating inflammatory responses is important to interpret the data obtained. The approach used to visualize and quantify the fate of NMs in the zebrafish will be reliant on what NMs are being investigated. For example, to image the fate of fluorescent NMs fluorescent microscopy can be used (e.g., confocal or light sheet microscopy) and for unlabelled NMs other imaging modalities will have to employed (e.g., transmission electron microscopy, CARS microscopy).

## Recommendations for new users

We have summarized the findings and experimental design employed in existing studies which have assessed neutrophil responses in zebrafish with a tail fin injury ( Table 1) and following the microinjection of different stimuli into the otic vesicle ( Table 2) in order to identify similarities and differences in the experimental design used. The tail fin injury model was selected as this has been widely used by different research groups in the published literature to assess inflammatory responses in transgenic zebrafish. Administration of substances via microinjection into the otic vesicle is the most popular route of exposure used in existing studies investigating inflammatory responses to different stimuli (and most commonly pathogens) following microinjection.

**Table 2.** Summary of the experimental design and findings of published studies investigating neutrophil responses in the otic vesicle following microinjection of the test substance or pathogen. Studies are presented in alphabetical order. The number of larvae included in each group is provided, as well as information on how many independent experiments were included, when available. The range of neutrophils counted in the otic vesicle is presented as well as the average count. When available data for the control group is included *.* The numbers of neutrophils accumulating following microinjection are not always explicitly stated in the text of research papers and so the number of neutrophils was extrapolated from the graphs presented and therefore may not be fully accurate.

Reference	Transgenic strain	Number of larvae/group (n)	Larvae age at injection (dpf)	Neutrophil count in otic vesicle (range and average)	Substance/pathogen microinjected	Hours post-injection when response was quantified
Buchan *et al*., 2019	Tg (lyz: MPO-mEmerald; lyz:nfsBmCherry) *Fluorescent neutrophils*	n = 25 (2 independent experiments)	3	5-54 (average: 21) 0-12 (average: 3)	Bacteria: *S. aureus* Control: PBS	4
Chen *et al*., 2021	Tg (mpx:eGFP) *Fluorescent neutrophils*	8	3	39-56 (average: 46), 23-40 (average: 34), 21-28 (average: 25) 0-4 (average: 1), 1, 0-4 (average: 2), 0-4 (average: 3)	Bacteria: *E. piscicida* Control: PBS	4, 12, 24
Deng *et al*., 2011	Tg (mpx:mCherry) *Fluorescent neutrophils*	36	3	4-65 Average: 32	Bacteria ( *Pseudomonas aeruginosa*)	1
Deng *et al.,* 2013	*Tg (mpx:dendra2)* *Fluorescent neutrophils*	9	3	0-26 (average: 8), 0-48 (average: 28), 2-55 (average: 38), 0-4 (average: 2), *neutrophils quantified in the head region	Bacteria ( *Pseudomonas aeruginosa (3 concentrations::* 25,000, 2500 CFU or bacteria number equivalent to 25000 CFU) Control: PBS	24
de Oliveira *et al*., 2013	Tg (mpx:gfp)i114 *Fluorescent neutrophils*	32 (3 independent experiments)	3	CXCL-8: 1-13 Average: 7 LTB4: 0-12 Average: 5 Control:0-5 Average: 2	PBS (Control), CXCL-8, LTB4	1
Gillies *et al.,* 2022	Tg (mpx: EGFP) ^i114^ *Fluorescent neutrophils*	24-36 (3 independent experiments)	3	Ag NMs: 1.95 μg/mL: 1-10 Average 6 Ag NMs: 3.9 μg/mL: 1-6 Average 5 AgNO3: 1.95 μg/mL: 1-7 Average 5 CXCL-8 (10nM): 2-7 Average 5 Control: 0 – 3 Average 2	Ag NMs CXCL-8 Control (PBS)	4,6,8,24,48
Harvie *et al*., 2013	Wild Type zebrafish *neutrophils imaged using Sudan black staining	11 – 16	3	10 CFU: 1-18 Average: 8 100 CFU: 6-20 Average: 15 1000 CFU: 10-40 Average: 20 Control: 0-9 Average:3	Bacteria ( *Streptococcus iniae*) @ 3 doses (10, 100, 1000 CFU) Control (PBS)	2
Lam *et al*., 2013	Not clearly reported: ( *Tg* ( *mpx:dendra2*) or *Tg* ( *mpx:mCherry*) *Fluorescent neutrophils*	Sample size not reported (although reported that 3 independent experiments performed)	3	4-30 (average: 15)	*Streptococcus iniae*	2
Powell *et al*., 2017	Tg ( *mpx*: Dendra) *Fluorescent neutrophils*	Up to 62	3	Bacteria: 16 Control: 3 *only averages presented	PBS (control), bacteria ( *P.* *aeruginosa*)	2

We observed a lot of variation in the design of existing studies, and we will focus on discussing how the following parameters may influence the experimental outcome: zebrafish strain and life stage selected, selection criteria for zebrafish embryos/larvae used in experiments, approach used to quantify the inflammatory response, sample size used, inclusion of positive and negative controls and dose selection.

### Transgenic strain selection

Various transgenic strains of zebrafish larvae can be used to assess inflammatory responses to NMs, with neutrophil accumulation most commonly investigated, and fewer studies also investigating macrophage responses. Several transgenic strains have been generated with fluorescently labelled neutrophils, however the Tg (mpx:eGFPi ^114^) strain has been the most popular strain of zebrafish used, to date ( Tables 1 and 2). Different strains may vary in their specificity (for neutrophils) and sensitivity, which will likely influence the magnitude of the inflammatory response that is activated. Whilst the Tg (lyz: EGFP) strain has been used to quantify neutrophil responses there is evidence that *lyz* is also expressed in macrophages and thus these strains may therefore not be suitable when assessing neutrophil responses ( Brannon *et al*., 2009, Hall *et al*., 2007, Keightley *et al*., 2014). Transgenic strains which exploit the myeloperoxidase (mpx) promoter (e.g. Tg mpx: EGFP) are therefore more commonly used as the mpx gene is neutrophil-specific ( Renshaw *et al*., 2006, Ruzicka *et al*., 2019). To investigate macrophage responses, the Tg mpeg:mCherry strain can be used ( Ellett *et al*., 2011). Investigating both neutrophil and macrophage responses in tandem is advantageous and can be achieved using the Tg mpx: EGFP/mpeg:mCherry strain ( Davis *et al*., 2016, Jim *et al*., 2016, Kaveh *et al*., 2020). There are also several methods than can be employed to deplete functional macrophages and neutrophils from zebrafish (reviewed by Rosowski, 2020) that may be useful when exploring the role of specific immune cell types in NM mediated inflammatory responses.

We have focused on the availability of transgenic strains that allow neutrophil and macrophage responses in zebrafish to be investigated. Other researchers may be interested in other immune cell types. For example, transgenic strains (e.g. *gata2:eGFP*) have been developed to investigate the role of eosinophils in inflammatory responses (e.g. Balla *et al*., 2010). The adaptive immune system does not develop until ~four weeks in zebrafish and thus the requirement to assess adaptive immune responses should inform what life stage of zebrafish is used. At this life stage the fish skin will no longer be transparent and thus the approaches discussed earlier to remove skin pigmentation will have to be used. Alternatively strains of zebrafish (with fluorescent immune cells) are available which are genetically modified to lack pigmentation and allow inflammatory responses to be monitored for a longer duration. Histology may also be performed to assess inflammatory responses in later life stages.

The strains already discussed are focused on investigating the accumulation of immune cells in specific sites following NM exposure, however other aspects of the inflammatory response may be of interest to explore using other transgenic strains. For example, the reporter strain Tg (NFκB:EGFP) could be utilised to assess the activation of the pro-inflammatory transcription factor NFκB ( Kanther *et al*., 2011). In addition, the activation of oxidative stress by NMs can be investigated via the visualization of the electrophile-response element (EpRE), a promoter region located on nuclear factor erythroid 2–related factor 2 genes (Nrf2), using the Tg (3EpRE:hsp70:mCherry) strain ( Mourabit *et al*., 2019). Furthermore, a strain with a hydrogen peroxide (H _2_O _2_) sensor has been developed and has been used to investigate the role of this reactive oxygen species (ROS) in leukocyte recruitment ( Niethammer *et al*., 2009).

### Embryo/larval screening criteria

Studies do not consistently report what criteria are used for zebrafish embryo/larvae selection. It is important to select healthy zebrafish embryos/larvae to ensure they are free from physiological abnormalities and that they are at the expected stage of development. Given the rapid rate of development of larval zebrafish, it is recommended that researchers ensure that life stages have been screened according to Kimmel *et al.* (1995) so that experimental cohorts are at the same developmental stages. If using transgenic strains it also imperative to ensure that they have a robust population of the fluorescently labelled cells of interest (e.g., macrophages and/or neutrophils). It is therefore important to select a consistent population of larvae to ensure obtained data are robust and comparable. Screening larvae to ensure that consistent numbers of the cells of interest are present can be done by visually assessing the larvae (using fluorescent microscopy) prior to the start of the experiment. However, this approach is subjective. Fluorescence based approaches, such as measuring the mean FI of the larvae using imaging software (e.g., Iplab or ImageJ) could be used to ensure the selected larval population fall within a user-defined range. Screening methods are not often well defined or reported currently in the published literature. It is therefore our recommendation that this information is provided in publications to ensure that data can be accurately compared between studies.

### Sample size

The sample size is the number of experimental units that have been included for each (control and treatment) group ( Lazic *et al*., 2018). However, the experimental unit is not always clearly reported in the published literature which use zebrafish embryos/larvae ( Tables 1 and 2). This is an important omission as if the experimental unit is not defined appropriately then the sample size can be over-estimated which can impact on the statistical analysis that is performed and interpretation of the data ( Lazic, 2022). As an example, for aqueous exposure, zebrafish embryos/larvae are typically exposed in microplates (e.g., 96, 24 or 12 well plates), but the number of larvae contained in each well is not often stipulated in published papers. If one zebrafish embryo/larva was added to each well and different treatments were added to different wells of the same plate, without cross-contamination between wells, then each zebrafish embryo/larva can be considered an experimental unit, and each embryo/larva contributes one data point to the analysis. However, if more than one zebrafish embryo/larva was added to each well then each well would represent an experimental unit as embryo/larva from the same well are not independent and cannot be allocated to different treatments. In this case, to avoid pseudoreplication, measurements taken from an individual embryo/larva can be averaged for each well so that each well provides one data point to the analysis ( Lazic, 2010). Researchers typically use <40 embryos/larvae for each (control or treatment) group ( Tables 1 and 2), and it is therefore assumed that independent experiments are performed, but it is unclear whether this is always the case and how many embryo/larvae are included in each experiment. In this case, using a block design would ensure that all treatment and control groups are included each time the experiment is performed, which would increase the chances of detecting an effect ( Festing, 2014). Furthermore, it is not common for researchers to indicate whether the position of control and treatment groups are randomized on a microplate. This is particularly important if there is potential for contamination of the test substance between wells. Therefore, it is recommended that researchers provide more detailed information on their experimental design when describing their methodology to ensure that the issues discussed above are addressed.

Power calculations can be performed to identify what sample size should be used but these are currently based on a limited data set. Therefore, when the model has been used more widely it is likely that more accurate estimations of the variability to be expected with this model will be available to calculate the sample size.

The number of zebrafish embryo/larvae that can be used in each experiment is limited by several factors such as; the number of fertilized embryos produced during spawning, the pre-screening process used to select zebrafish embryos/larvae for inclusion in the study (as this dictates how many embryos/larvae are available to the researcher), the time taken to perform the experiment (e.g., anaesthetizing larvae, inducing a tail fin injury or microinjecting larvae, acquiring images, and larval recovery time), the number of treatment and control groups desired, and the number of researchers performing the experiment (as this will impact on equipment availability). These limiting steps should be taken into consideration when designing experimental work to ensure it is feasible. For example, imaging time points should be set at practical intervals to reflect the time needed to acquire images of all the larvae in all treatment and control groups.

### Life stage

In zebrafish larvae, macrophages are the first immune cells to appear and which occur from 22 hpf ( Rosowski, 2020) and neutrophils are apparent from 2 dpf ( Lieschke *et al*., 2001). Therefore, when assessing inflammatory responses in larval zebrafish, experiments can start from 2 dpf. However, at this stage the chorion is still present and must be removed either manually or chemically to ensure exposure of the zebrafish to the NMs. Dechorionating embryos is time consuming and can damage the larvae (i.e., the process itself can cause inflammatory response that may interfere with the experiment). Thus, most studies performed to date ( Tables 1 and 2) have exposed zebrafish after they have hatched.

For the tail fin injury model and for microinjection studies using the otic vesicle the age of zebrafish used is typically 3 dpf ( Tables 1 and 2). The zebrafish larvae are non-protected until 5 dpf, thus starting at 3 dpf allows for inflammatory responses to be monitored for up to 48 hours in non-protected life stages. Using zebrafish at this life stage also means that zebrafish will have hatched from the chorion naturally prior to beginning experiments. The use of later time points would require the use of protected life stages of zebrafish which would have more ethical implications and require permission from responsible authorities. However, the duration of the experiment will be limited by the transparency of the fish, as it becomes more challenging to monitor the inflammatory response as skin pigmentation increases (see above).

### Quantification of the immune response

To assess whether an inflammatory response is activated in zebrafish, the accumulation of immune cells to the target site of interest needs to be visualized and quantified. Histological examinations have been used to visualize inflammatory responses to NMs in wild type zebrafish strains, however, here we focus on how to quantify responses in transgenic zebrafish strains with fluorescently labelled immune cells. In order to quantify inflammatory responses, images are typically taken in the same larvae at different time points using fluorescent microscopy (e.g., stereomicroscopy, confocal microscopy, light sheet microscopy) and immune cells are counted in the region of interest (e.g. injury or injection site and/or whole organism). Depending on the target site of interest, imaging modalities that enable long-term imaging without photo-bleaching and permit 3D image reconstruction may be employed such as selective plane illumination microscopy (SPIM) ( Taylor *et al*., 2019). Several published studies have investigated responses at only one time point ( Tables 1 and 2). However, to capture the initiation, peak, and resolution (or lack thereof) of inflammation in non-protected life stages of zebrafish, it is recommended that multiple time points are used as this allows the dynamics of the inflammation response to be investigated. When imaging at set time points larvae are most commonly placed on a glass microscope slide (in the presence or absence of agarose) to capture the required image. The use of microfluidic devices may also be useful when imaging discrete regions such as the otic vesicle, as these enable the immobilisation of the larvae in the desired position (e.g., Bischel *et al.* 2013, Ellett and Irimia, 2017a, 2017b). It is recommended that images of the whole larvae as well as the region of interest (e.g., otic vesicle, swim bladder, gill, injury site) are taken as the NMs may elicit a local and systemic inflammatory response ( Gillies *et al*., 2022). In addition, if possible, it would be useful to take images at different focal planes (z stacks) to ensure the images capture cells present in three dimensional regions of interest such as the otic vesicle or swim bladder, which would require access to confocal or light sheet microscopy (e.g., Logan *et al*., 2018, Winter *et al.,* 2017).

The accumulation of cells can be quantified by performing manual or automated counts, often using software such as Fiji (ImageJ) or Imaris. Manually counting cells is time consuming and more subjective, however, it is a commonly used technique when quantifying cellular responses in zebrafish larvae ( Loynes *et al*., 2010, Miskolci *et al*., 2019, Gratacap *et al*., 2014). The error involved in manual counting can be reduced by having at least two people to quantify the responses to confirm findings, and via experimental blinding, such as the method described by d’Alençon *et al*., (2010). Automated software can be exploited to facilitate rapid and accurate cell counts, such as cell counting macros/plugins that can be used in software such as ImageJ (e.g. Renshaw *et al.,* 2006, Renshaw and Ingham, 2010, Wittmann *et al.,* 2012). Automated counting requires validation (e.g., the user should perform some manual counts to confirm the data), however existing studies have shown good correlation between automated and manual counts ( Renshaw *et al.,* 2006). Alternatively, the inflammatory response can be quantified via fluorescence intensity-based approaches. For example, by measuring the fluorescence intensity (FI) of the region of interest rather than counting individual cells, an increase or decrease in FI can be used to indicate an increase or decrease in the numbers of immune cells (e.g. Duan *et al.,* 2016).

Whilst imaging at set time points is useful for the quantification of the inflammatory response, it may be advantageous to acquire time-lapse video imaging which has the added benefit that it can enable the tracking of immune cells ( Feng *et al.,* 2010, Kaveh *et al*., 2020). To maintain the zebrafish larvae in a specific position for the duration of time-lapse imaging, they can be mounted on glass microscope slides using low melting point (LMP) agarose (e.g. Hirsinger and Steventon, 2017). This technique is not appropriate for long periods of time, as it requires the use of a heated microscope stage to allow for normal embryo development, and the agarose may have to be replaced/adjusted throughout the experiment to accommodate growing larvae. Guidance is available in the published literature on how to overcome this issue (e.g. Hirsinger and Steventon 2017).

### Dose selection

Zebrafish embryos/larvae have been used widely to assess NM (eco) toxicity, as such toxicity data (i.e., mortality, growth, development) for many NMs have been published, which can inform the selection of non-lethal doses for immune effect directed studies. For example, LC _50_ values (the concentration required to kill 50% of the exposed population) are known for NMs from the JRC repository (which are widely studied by the international scientific community) including for example Ag (e.g. NM300K) and ZnO (e.g. NM110) ( Massarsky *et al*., 2013, Muth-Köhne *et al*., 2013, Osborne *et al*., 2013, Küçükoğlu *et al*., 2013, Choi *et al*., 2016). When using transgenic zebrafish larvae to assess inflammatory responses to NMs of known toxicity, appropriate sub-lethal concentration ranges can therefore be determined from the existing data derived from aquatic toxicology studies using wild type zebrafish embryos/larvae. If data are not available for the NM of interest then it is advised that dose finding studies should be performed as part of pilot work.

### Negative and positive controls

When working with zebrafish larvae it is difficult to maintain a sterile environment. It is therefore possible that the inflammatory responses in larvae may be influenced by contaminants (e.g., endotoxin (LPS)) present in the exposure medium. However, existing studies often neglect to report what controls are included. Brown *et al*. (2007), found that in the absence of an infectious agent, a tail-nick wound would not elicit a moderate inflammatory response. This study highlights a need to include appropriate negative controls and to better monitor the presence of microbes and their products within the system water from which the larvae are cultured as such factors may affect leukocyte recruitment. The microbial environment must therefore be acknowledged as a potential confounding variable which has a potential impact on aspects of the immune response such as recruitment and cytokine expression.

The inclusion of positive controls demonstrates that you are able to detect the response of interest (e.g., inflammation) within your test model. We assessed inflammatory responses to several positive controls in both the tail fin injury and otic vesicle microinjection experiments ( Gillies *et al*., 2022) as we observed that existing studies neglected to consistently include a positive control. More specifically, we evaluated what inflammatory response were activated by the pro-inflammatory compound LPS and the chemoattractants fMLF, LTB _4_, C5a, and CXCL8. Our findings suggested that the positive control required will depend upon the route of exposure ( Gillies *et al*., 2022). For example, LTB _4_ was identified as the most appropriate positive control for tail fin injury model and CXCL8 was the most effective substance at stimulating an inflammatory response following microinjection into the otic vesicle.

For aqueous exposure of injured fish, NMs are typically suspended in zebrafish water of various types (e.g., E3, OECD medium). It is therefore essential to include zebrafish medium as a negative control to quantify the inflammatory response in the absence of NMs (or the test substance of interest). As the microinjection itself may cause an injury that stimulates an inflammatory response, it is crucial to include an appropriate negative and vehicle control (e.g., PBS in the absence of the test substance). For comparative purposes it is also useful to include a group with has not been injected.

## 3Rs benefits

A PubMed search (Oct 2022, search terms: nanomaterial OR nanoparticle AND lung AND rat or mouse AND inflammation AND neutrophil) revealed that >300 rodent studies have assessed pulmonary inflammatory responses to NMs, with a higher proportion of existing studies using mice as the test model. Such studies typically assess the toxicity of a panel of NMs (average of three, ranging from one to 13), administer more than one dose (average of two, ranging from one to seven) and assess inflammation at several time points (average of three, ranging from one to six). The average group size used for mouse studies is eight (with an average of 75 animals used/study), and for rats the average group size is six (with an average of 65 animals used/study). Existing studies have typically used only one sex of rodents, although some studies have used both male and female animals (e.g., Wan *et al*., 2017, Srinivas *et al*., 2012). In addition, one route of administration is typically used, with intratracheal instillation being prioritized, although some studies have compared the response observed following two routes of exposure (e.g. intratracheal instillation and inhalation ( Morimoto *et al.,* 2016, Silva *et al*., 2014)). The variety of NMs currently in use or development in addition to the diversity of future generations of NMs means there are a large number (tens of thousands) of NMs whose safety will need to be assessed. Therefore, it is not sustainable to rely on rodent testing for nanotoxicology testing and we suggest that the increased use of zebrafish could offer many 3Rs benefits. For example:

i) Non protected life stages of zebrafish could be used to screen NM toxicity as an alternative to using rodents leading to a **replacement** of animal (rodent) use. For example, tests performed using early life stages of zebrafish could identify highly toxic NMs at an early stage of innovation whose toxicity would not be tested further in rodents.
ii) Non protected life stages of zebrafish could be used as a bridge between *in vitro* and rodent studies to help inform the design of rodent studies and more specifically to:
  a. Help prioritize NM selection for more extensive testing in rodent models, thereby leading to a **reduction** in rodent use. This is particularly important as nanotoxicology rodent studies frequently test the toxicity of a panel of NMs and data obtained from zebrafish could be used to prioritise NM selection for *in vivo* (rodent) testing.
  b. Allow testing of fewer doses of each NM in rodents, leading to a **reduction** in rodent use as data from a whole organism (zebrafish) has already been obtained to investigate NM toxicity.
  c. Help **refine** rodent studies as high toxicity NMs could be identified and this knowledge can inform dose selection for rodent studies to avoid administration of highly toxic doses to rodents.
iii) Inflammatory responses can be monitored in the same organism over time leading to a **reduction** in animal use as separate treatment and control groups are not required for each time point of interest.

In the short term we anticipate that the use of zebrafish will precede rodent work to identify whether rodent testing is needed and to help inform the experimental design of rodent studies. This would require that a tiered testing strategy is employed to assess NM toxicity. Such testing strategies would progress from applying simple and advanced *in vitro* models, before employing (non-protected life stages of) zebrafish and finally rodent testing would only be performed if deemed essential e.g., for regulatory approval (e.g., Johnston *et al*., 2018, Stone *et al*., 2020). The use of zebrafish in nanotoxicology is still in its infancy. Once the model has been adopted more widely, and standard protocols have been developed that allow testing to be performed in a harmonized manner, it is possible that zebrafish may replace rodent testing when investigating NM mediated inflammatory responses (as an indicator of their toxicity) in the longer term.

We have focused on how zebrafish can be applied to assess inflammatory responses to NMs to screen their toxicity but inflammatory responses to other substances could be investigated in this model and other transgenic strains could be used to assess other adverse responses to NMs.

## Conclusions

Investigation of the capacity of NMs to stimulate an inflammatory response is commonly used to screen their toxicity in *in vitro* and *in vivo* (rodent) models. Transgenic zebrafish offer an effective alternative (i.e., non-mammalian) model to assess whether NMs activate inflammatory responses when assessing their safety. Indeed, we have successfully used non-protected life stages of transgenic zebrafish with fluorescent neutrophils (e.g., Tg (mpx: EGFP ^114^) to investigate inflammatory responses to NMs and thereby demonstrated their usefulness as a model for evaluating NM toxicity. More studies are now required to test a wider panel of NMs, to explore more administration routes and to assess the utility of other transgenic strains.

We have outlined how zebrafish may be exposed via water or microinjection to NMs to reflect exposure routes that are relevant to human/rodent exposure. As not all systems that are relevant to human and rodent exposure are fully developed in non-protected life stages of zebrafish the use of a wider range of exposure routes has been explored as their use provides an opportunity to make testing more ethical. More specifically, we have highlighted exposure routes that can be employed in non-injured or injured non protected life stages of zebrafish to screen NM toxicity. The experimental design employed in existing studies to investigate inflammatory responses to a range of pathogens and stimuli in zebrafish is diverse and can have a profound impact on the experimental outcome. It is therefore essential for researchers to justify their approach and to provide sufficient detail on the experimental design employed so that data can be interpreted correctly and to allow comparisons to be made between studies. Ideally, standard methods will be developed that allow the inflammatory response activated by NMs (and other chemicals, pharmaceuticals and pathogens) to be assessed in a consistent manner The increased use of zebrafish in nanotoxicology is likely to enhance implementation of the 3Rs principles to make testing more ethical, cheaper, quicker and potentially more predictive. Whilst a focus has been placed on the use and application of transgenic zebrafish for assessment of pro-inflammatory responses activated by NMs in this paper the themes discussed are not restricted to NMs but relevant also to their appliction in ecotoxicology or human health focused studies investigating other substances (e.g. chemicals, pharmaceuticals) and pathogens as well as for studies investigating disease pathogenesis.

## Data Availability

No data are associated with this article.
