# Supplementary material for: Application of transgenic zebrafish for investigating inflammatory responses to nanomaterials: Recommendations for new users
Source: F1000Res. 2025 Jun 23;12:51. Originally published 2023 Jan 12. [Version 2] doi: 10.12688/f1000research.128851.2 (PMC12255896; doi:10.12688/f1000research.128851.2)
Supplement: Peer review history

# Reviewer report: Reviewer response for version 2

**DOI:** 10.5256/f1000research.183735.r394059

**Published:** 2025-07-12

**Authors:** Stephen Renshaw, Catherine Loynes

**Affiliations:** 1 University of Sheffield, Sheffield, UK

We are happy with the amendments that have been made and the authors have addressed our previous comments thoroughly and clearly. There are still inconsistencies to the zebrafish transgenic lines nomenclature but this doesn't impede the understanding of the article.

Is the review written in accessible language?

Yes

Are all factual statements correct and adequately supported by citations?

Yes

Are the conclusions drawn appropriate in the context of the current research literature?

Yes

Is the topic of the review discussed comprehensively in the context of the current literature?

Yes

Reviewer Expertise:

NA

We confirm that we have read this submission and believe that we have an appropriate level of expertise to confirm that it is of an acceptable scientific standard.

# Reviewer report: Reviewer response for version 1

**DOI:** 10.5256/f1000research.141482.r327733

**Published:** 2024-10-11

**Authors:** Sijie Lin

**Affiliations:** 1 College of Environmental Science and Engineering, Biomedical Multidisciplinary Innovation Research Institute, Shanghai East Hospital, Tongji University, Shanghai, China

This is a timely and comprehensive report on using transgenic zebrafish for immune response to nanomaterials. Just a couple suggestions for the authors to consider.

 1. The use of zebrafish to "enhance the implementation of the 3Rs principles" may still be up for debate? As zebrafish, especially after 4-5 days, is considered as an intact animal.

 2. On Scientific benefit: "Zebrafish could provide better predictions of the impact of substances on human health than rodents", "better predictions" might be a bit too exaggerated, faster or quicker would be better.

 3. Reports using transgenic zebrafish to understand the immune responses of engineered nanomaterials are recommended for the authors to consider [Ref-1], [Ref-2].

Is the review written in accessible language?

Yes

Are all factual statements correct and adequately supported by citations?

Yes

Are the conclusions drawn appropriate in the context of the current research literature?

Yes

Is the topic of the review discussed comprehensively in the context of the current literature?

Yes

Reviewer Expertise:

Zebrafish toxicology, nanotoxicology

I confirm that I have read this submission and believe that I have an appropriate level of expertise to confirm that it is of an acceptable scientific standard.

## References

[1] : Environmental Hazard Potential of Nano-Photocatalysts Determined by Nano-Bio Interactions and Exposure Conditions. *Small* .2020;16(21) : 10.1002/smll.201907690 e1907690 10.1002/smll.201907690 32191389

[2] : Redox Activity and Nano-Bio Interactions Determine the Skin Injury Potential of Co3O4-Based Metal Oxide Nanoparticles toward Zebrafish. *ACS Nano* .2020;14(4) : 10.1021/acsnano.9b08938 4166-4177 10.1021/acsnano.9b08938 32191835

# Reviewer report: Reviewer response for version 1

**DOI:** 10.5256/f1000research.141482.r326092

**Published:** 2024-10-11

**Authors:** Stephen Renshaw, Catherine Loynes

**Affiliations:** 1 University of Sheffield, Sheffield, UK

This review summarises different approaches of exposure within zebrafish to investigate the inflammatory response to nanomaterials (NM). It is specifically aimed at researchers new to using zebrafish. The authors provide a good introduction to using zebrafish as a model organism and how it can contribute to the 3Rs. They give essential detail on key aspects to think about when using the zebrafish as a model and screening tool that will promote experimental design discussions amongst new users. The review has 2 main sections: it addresses NM delivery and then more specifically, how to study the inflammatory response. Some transgenic lines are mentioned that are key to investigating the inflammatory response *in vivo* but there are several missing (see sections below).  The language used is clear and accessible and the review uses nice diagrams to summarise information. Occasionally, some paragraphs are very brief and feel oddly placed, without a connection or explanation as to why the statements are there.

 The information given is accurate and mainly cited correctly and thoroughly. Other reference suggestions are listed below.

References

 Some key references are missing as detailed below:

*Section: Zebrafish and Inflammation*

 Page 4, 1 ^st^ paragraph – 2 groups first identified granulocytes in zebrafish around the same time and both should be cited.

 Lieschke GJ, et al., 2001 (Ref 1)

 Bennett CM, et al., 2001 (Ref 2)

 Page 5 – Include fms: GFP line (Dee, 2016) and mfap4 lines (Walton 2016) as alternatives to mpeg lines to study macrophages. Both are useful depending on what aspect of macrophages the researcher is investigating.

 Dee CT, et al., 2016 (Ref 3)

 Page 22. Reference for automated analysis is from the Grabher group not Renshaw and Ingham 2010, which is a secondary source.

 d'Alençon CA, et al., 2010 (Ref 4)

Nomenclature

 Our own early manuscripts did not use correct nomenclature and we are grateful to the reviewers who pointed us in the right direction. The nomenclature of the transgenic lines is not correct in this review (according to zfin nomenclature conventions) i.e. all italics, state if a BAC was used, allele numbers should not be superscript. Allele numbers are not included for many of the lines suggested and as this review is directed at new users it would be very informative to include these. E.g. *TgBAC(mpx:GFP)i114*

 In the legend for Figure 1, this compound transgenic should be *TgBAC(mpx:GFP)i114; Tg(mpeg:mCherry) *-adding the allele code for the mpeg line in italics. This approach should be used for all transgenics, as set out by ZFIN nomenclature conventions.

Section details:

*Research highlights.*

 “Zebrafish could provide better predictions of the impact of substances on human health than rodents” No evidence is provided for this model being better than rodents. It seems likely there are some ways that this model might have advantages (for example 3Rs) but others (tissue specificity etc) where rodents might be more beneficial. I would suggest a more nuanced discussion.

*Introduction*

 Page 4. “In the EU, early life stages of zebrafish, prior to exogenous (independent) feeding at 120 hours/5 days post fertilisation (hpf or dpf)), are not protected by law and as such do not require permission from responsible authorities to be used in scientific research (EU Directive 2010/63/EU).” I’m not clear this is completely correct. Although the larvae are not “protected” under UK law, their transgenic parents are, meaning that the experiments *are* protected by law. I would also consider the generation of a genetically modified larvae to be a regulated procedure. I would suggest this paragraph is revised after appropriate discussion.

*Nanomaterials and inflammation*

 Page 6, second sentence would be useful to have a reference for this where it is stated that the properties exhibited at nanoscale differ drastically to larger forms.

*Zebrafish and nanotoxicity*

 Page 7, 2 ^nd^ paragraph. I think more needs to be explained as to why transgenic fish are advantageous for this specific study of NMs, over the WT fish stated in the first paragraph, especially in terms of studying the inflammatory response. If the Ecotoxicity and Inflammation sections were combined on page 7 it would resolve this issue and flow better. The first 2 paragraphs seem disjointed so a rewrite of the 3 paragraphs together would make this section clearer.

*Aqueous exposure*

 Page 9, first paragraph – talks about limitations of increased pigment development. Could you talk about nacre or casper at this point as an option?

 Zebrafish gills are quite unlike human lungs in many ways. For balance a discussion of the differences to human lungs would be helpful here.

*Tailfin injury model*

 Page 12, 5 ^th^ paragraph, reference Elks et al 2011 doesn’t show that the severity of injury inflicted influences the inflammatory response that is activated. The same reference is also used to cite the method of a small nick in the tail fin. Again, this isn’t in this ref. This paper isn’t cited in Table 1 either.

Layout/order

 The section starting on page 8 to page 11 (Selection of Exposure Route) doesn’t flow. The section of Other Exposure Routes really could fit under the other main headings.

 Pulmonary exposure

                      Aqueous

                      Microinjection – swim bladder…add otic vesicle and hindbrain here.

 Oral exposure

 Dermal exposure

 Intravenous exposure

 Inclusion of ‘Tail fin injury model’ in this subsection of ‘other routes of exposure’ doesn’t fit. This should be its own section about how to monitor the inflammatory response following one or more of these exposure routes.

 Yolk sac injection is included within the section on intravenous exposure, which is incorrect.

Is the review written in accessible language?

Yes

Are all factual statements correct and adequately supported by citations?

Yes

Are the conclusions drawn appropriate in the context of the current research literature?

Yes

Is the topic of the review discussed comprehensively in the context of the current literature?

Yes

Reviewer Expertise:

Zebrafish models of innate immunity

We confirm that we have read this submission and believe that we have an appropriate level of expertise to confirm that it is of an acceptable scientific standard, however we have significant reservations, as outlined above.

## References

[1] : Morphologic and functional characterization of granulocytes and macrophages in embryonic and adult zebrafish. *Blood* .2001;98(10) : 10.1182/blood.v98.10.3087 3087-96 10.1182/blood.v98.10.3087 11698295

[2] : Myelopoiesis in the zebrafish, Danio rerio. *Blood* .2001;98(3) : 10.1182/blood.v98.3.643 643-51 10.1182/blood.v98.3.643 11468162

[3] : CD4-Transgenic Zebrafish Reveal Tissue-Resident Th2- and Regulatory T Cell-like Populations and Diverse Mononuclear Phagocytes. *J Immunol* .2016;197(9) : 10.4049/jimmunol.1600959 3520-3530 10.4049/jimmunol.1600959 27694495 PMC5073357

[4] : A high-throughput chemically induced inflammation assay in zebrafish. *BMC Biology* .2010;8(1) : 10.1186/1741-7007-8-151 10.1186/1741-7007-8-151 21176202 PMC3022775
